# Evaluation of carboxyfluorescein-labeled 7-methylguanine nucleotides as probes for studying cap-binding proteins by fluorescence anisotropy

**DOI:** 10.1038/s41598-021-87306-8

**Published:** 2021-04-08

**Authors:** Anna Wojtczak, Renata Kasprzyk, Marcin Warmiński, Krystian Ubych, Dorota Kubacka, Pawel J. Sikorski, Jacek Jemielity, Joanna Kowalska

**Affiliations:** 1 Division of Biophysics, Institute of Experimental Physics, Faculty of Physics, University of Warsaw, Ludwika Pasteura 5, 02-093 Warsaw, Poland; 2 Centre of New Technologies, University of Warsaw, Stefana Banacha 2c, 02-097 Warsaw, Poland; 3 College of Inter-Faculty Individual Studies in Mathematics and Natural Sciences, University of Warsaw, Stefana Banacha 2c, 02-097 Warsaw, Poland

**Keywords:** Molecular biophysics, Biochemistry, Drug screening, Bioanalytical chemistry, Fluorescent probes

## Abstract

Fluorescence anisotropy (FA) is a powerful technique for the discovery of protein inhibitors in a high-throughput manner. In this study, we sought to develop new universal FA-based assays for the evaluation of compounds targeting mRNA 5′ cap-binding proteins of therapeutic interest, including eukaryotic translation initiation factor 4E and scavenger decapping enzyme. For this purpose, a library of 19 carboxyfluorescein probes based on 7-methylguanine nucleotides was evaluated as FA probes for these proteins. Optimal probe:protein systems were further investigated in competitive binding experiments and adapted for high-throughput screening. Using a small in-house library of compounds, we verified and confirmed the accuracy of the developed FA assay to study cap-binding protein binders. The applications of the most promising probes were then extended to include evaluation of allosteric inhibitors as well as RNA ligands. From this analysis, we confirmed the utility of the method to study small molecule ligands and evaluate differently 5′ capped RNAs.

## Introduction

A 7-methylguanosine cap structure is present at the 5′ end of eukaryotic mRNA and influences numerous cellular functions related to mRNA metabolism^1^. 7-Methylguanosine (m^7^G) is a positively charged nucleoside that, together with the negatively charged 5′,5′-triphosphate chain linking it to the first transcribed nucleotide of RNA, creates a unique molecular recognition pattern targeted by specific proteins involved in mRNA turnover^2,3^. Cap-protein interplay is crucial for gene expression processes, such as pre-mRNA splicing, transport, translation, and degradation^4–6^.

One of the main cap-binding proteins is eukaryotic initiation translation factor 4E (eIF4E). Recognition of the cap structure by eIF4E is the rate-limiting step during translation initiation^7^. The active pool of eIF4E is highly regulated in healthy cells^8^. In contrast, eIF4E is often overexpressed in cancer cells, thereby promoting cell growth and survival^9^. Overexpression of eIF4E results in increased translation of mRNAs encoding oncoproteins and growth factors^10^. Reduction of eIF4E levels is not detrimental for normal mammalian physiology, therefore, it creates an opportunity for therapeutic targeting of eIF4E to selectively inhibit oncogenic translation^11^. Hence, identifying new high-affinity ligands to limit active pools of eIF4E is the first step towards the development of therapeutic strategies in anticancer treatment^12,13^.

Another cap-binding protein is decapping scavenger enzyme (DcpS), which prevents the accumulation of free cap structures released as a result of 3′-to-5′ mRNA decay, thereby blocking inhibition of proteins crucial for mRNA splicing and translation and avoiding potentially toxic effects^14^. DcpS plays also a more general role in the control of gene expression and has been independently linked to spinal muscular atrophy^15^, intellectual disability^16^, cancer^17^, and microRNA processing^18^. DcpS is a therapeutic target in spinal muscular atrophy (SMA), an autosomal recessive disease caused by deletion or mutational inactivation of the survival motor neuron (*SMN*) 1 gene^19^. Inhibition of DcpS by C5-substituted quinazolines has been shown to activate *SMN2* gene expression in vitro, although the mechanism of this activation has not yet been fully elucidated^15^. Furthermore, studies performed in SMA model mice have shown therapeutic effects, such as prolonged survival and improved motor function^20^. In 2015, the loss of DcpS enzyme activity was connected with a novel clinical entity referred as Al-Raqad syndrome (ARS)^16^. ARS is caused by homozygous or heterozygous mutations resulting in loss-of-function alleles in the *DcpS* gene^21^ and is associated with severe growth delay, neurological defects, and skeletal and cardiac anomalies. DcpS has also been shown to be essential for the survival of acute myeloid leukemia (AML) cells^17^. Accordingly, DcpS has become a drug discovery target^15,22^.

Molecules targeting eIF4E or DcpS can act as modulators (activators or inhibitors) of various processes involving these cap-binding proteins; therefore, these modulators are potential therapeutics and useful research tools. A commonly used technique to study interactions between cap-binding proteins and ligands is fluorescence quenching titration (tsFQT)^3^. However, this approach is time-consuming, requires relatively high protein concentrations, is low throughput, and has other methodological limitations, which make it unsuitable for drug discovery applications. Therefore, it is necessary to develop higher throughput yet accurate methods for the discovery of ligands targeting cap-binding proteins. Fluorescence polarization (FP) and fluorescence anisotropy (FA) have been previously employed to study eIF4E-ligand interactions, yielding different outcomes^23–25^. Although several m^7^G analogs were developed as probes for FP, a systematic study of structure–activity relationships has never been performed. In recent years, fluorescent methods based on m^7^G analogs have been optimized to measure DcpS activity^26–29^. These methods are based on fluorescently labeled or fluorogenic substrates to measure reaction progress and have been successfully adopted for inhibitor evaluation^30–32^. DcpS-binding ligands have also been studied using other methods, including high-performance liquid chromatography^33^, microscale thermophoresis^34^, tsFQT^35^, and radioactive assays^15^.

In this work, we synthesized and evaluated a library of different fluorescently labeled cap analogs as probes for the development of an FA approach for the discovery of molecules targeting DcpS or eIF4E. In order to find an optimal probe, the probe set included compounds differing in size, modification sites, and the presence of additional modifications within the triphosphate bridge (imidophosphate, methylenobisphosphonate, phosphorothioate, or phosphorothiolate). The binding affinities of the probes to DcpS and eIF4E were characterized using FA, and the most suitable probes were then used for the development of an FA method that was adaptable to high-throughput screening conditions. The developed methods were verified using a small in-house library of nucleotide derivatives. For selected ligands, the half-maximal effective concentration (EC_50_) was determined and compared with binding affinities obtained by alternative methods. Overall, our findings showed that the established method could be used to study nucleotide-derived ligands (including oligo-RNA) and other compounds targeting cap-binding proteins with high accuracy. Furthermore, we demonstrated that the method could also be used to study allosteric binding of eIF4E.

## Results and discussion

### Optimization of the probe and binding studies

**Figure 1:**
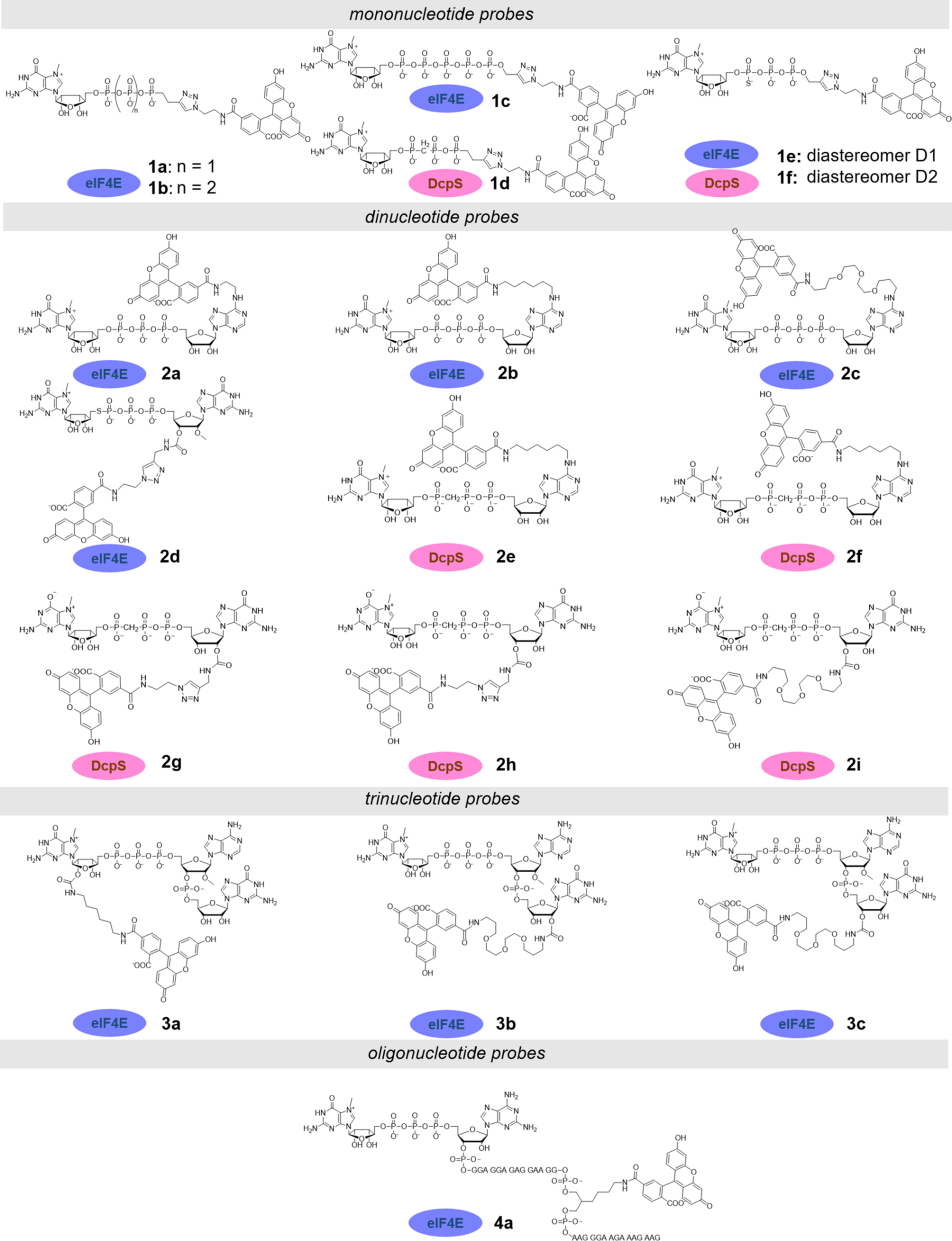
Structures of FA probes for cap-binding proteins evaluated in this work.

As the initial step in the development of an FA method, different structures of fluorescent probes were explored. As a starting point for the design of the probes, we used several known cap-derived eIF4E and DcpS binders differing in structural complexity (Fig. 1). As a label, we chose carboxyfluorescein (FAM) because of its many advantages in the context of FA assays, including high quantum yield and short half-life of the excited state (~ 4 ns)^36^, which is beneficial for small molecular probes^37^. As a result, we synthesized and tested a set of carboxyfluorescein-labeled cap analogs differing in size (from mono- to trinucleotides) and the site of fluorophore attachment (Fig. 1, Fig. S1). The fluorophore was attached using different chemical strategies to either the terminal phosphate, the 2′ or 3′ hydroxyls of m^7^G or G ribose moiety, or the *N*6-position of adenine. Fluorescent probes for studies with DcpS were additionally modified within the triphosphate bridge to make them resistant to enzymatic hydrolysis. To this end, different phosphate modifications were explored, including a bridging modification (β-γ-O to CH_2_)^38^, nonbridging modification (γ-O-to-S)^39^, and a recently reported phosphorothiolate modification (5′-PSL)^30^. As a reference, we included a 30-nt long capped-RNA probe that was previously used for binding studies with *Drosophila melanogaster* eIF4E^40^. This probe could be considered a mimic of the natural ligand of eIF4E (mRNA), wherein the probe was placed 16 nt away from the 5′ end, thereby minimizing its impact on protein binding.

**Figure 2:**
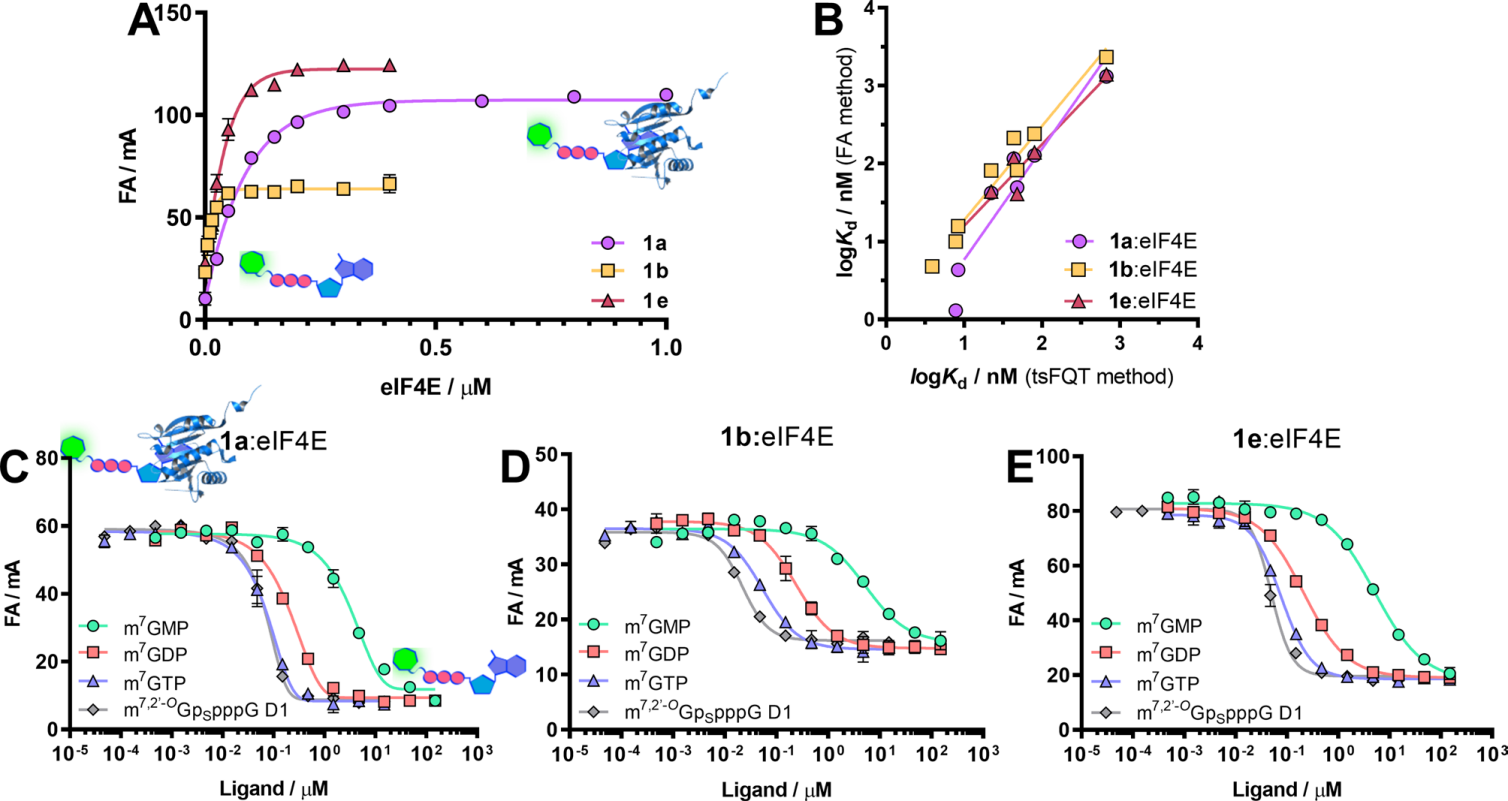
Development of an FA binding assay for eIF4E. (**A**) Binding curves for probes **1a**, **1b**, and **1e** with eIF4E protein. (**B**) Correlation between log*K*_D_ values for seven ligands obtained by the FA method with the use of probes **1a**, **1b**, and **1e** and log*K*_D_ values determined by tsFQT. (**C**–**E**) Dose–response curves obtained from FA competition assays for the four selected ligands using probes **1a** (**C**), **1b** (**D**), and **1e** (**E**). The protein concentration in the competition experiment was set above the *K*_D_ value to achieve 70–85% of the maximum response. Dose–response binding experiments were carried out with a serial half-log dilution of unlabeled ligands. Data shown are mean values ± standard deviations of three independent experiments, each performed in duplicate.

All the fluorescently labeled compounds were evaluated as FA probes for eIF4E and/or DcpS proteins. The optimal probe fulfilling the requirement for unbiased *K*_D_ estimation and development of competition binding assays should have high affinity for the target protein and a stable intrinsic fluorescence intensity that remains unchanged over time and upon binding to the target protein^41,42^. To select optimal probes, we performed direct binding experiments, in which each probe (at a constant concentration) was mixed with increasing concentrations of eIF4E or DcpS. We also performed negative control experiments for select probes using Bovine Serum Albumin (BSA) to confirm lack of unspecific interactions at concentrations up to 2.5 µM (Fig. S2). To check whether the emission of the ligand changed upon binding to the specific protein, values of total intensities were calculated as the sum of the parallel and double perpendicular intensities for each binding experiment^43,44^. Probes were compared based on the brightness enhancement factor *g*, demonstrating enhancement of total intensity between the free and bound forms of the probe. We observed that the changes in emission intensity during protein-probe complex formation strongly depended on the site of cap analog labeling and the linker length (Table 1). For both tested proteins, the greatest changes in fluorescence intensity were observed when the label was located at the ribose moiety. Compounds with the label at the 2′ position were more sensitive to environmental changes than those labeled at the 3′ position (a 1.2-fold difference for probes **3b** and **3c**). In contrast, cap analogs labeled at the *N*6 position of adenine had the most stable fluorescence signal. These dependencies changed with modifications within the phosphate bridge (e.g., the additional phosphate group in probe **1c** decreased the fluorescence intensity stability in comparison with probes **1a** and **1b**). The unfavorable effects of binding-sensitive fluorescence intensity could be successfully eliminated by changing the length of the linker (Table 1). For DcpS protein, longer linkers were associated with more stable fluorescence intensity (probe **2i** was 1.4 times less sensitive than probe **2h**). In the case of eIF4E, the smallest changes in fluorescence intensity were observed for the medium-length linker (the intensity change for probe **2b** was 4%, whereas those for probes **2a** and **2c** were 10% and 9%, respectively). The stability of fluorescence intensity was also affected by FAM regioisomerism; in binding studies with DcpS, isomer 5 of FAM led to significantly greater changes in fluorescence intensity than isomer 6. To determine the dissociation constant (*K*_D_) values, a 1:1 binding model was fitted to the obtained binding curves (Table 1, Fig. 2A). For probes characterized by Δg values greater than 0.1, the FA values were appropriately corrected before *K*_D_ determination^44^. Among fluorescent probes tested against eIF4E, mononucleotide cap analogs (**1a**, **1b**, **1c**, **1e**, **1f**) bound the protein with significantly higher affinity than other probes. The affinity for eIF4E was the highest for mononucleotide analogs carrying a tetraphosphate chain (compound **1b** with a *K*_D_ that was 5.7-fold lower than that of the triphosphate probe **1a**). Despite the high affinity for eIF4E, probe **1b** showed the lowest FA response upon transition from the free to bound state, which affected method quality. Interestingly, further elongation of the tetraphosphate bridge to pentaphosphate did not improve the binding. The affinity of the probes containing phosphorothioate modification (**1e**, **1f**) to eIF4E was dependent on the absolute configuration of the stereogenic P center (probe **1e** bound to eIF4E 1.5 times stronger than probe **1f**), consistent with previous data reported for unlabeled compounds^39^. Phosphorothioate substitution improved the binding compared with unmodified probe (**1e** showed a *K*_D_ 1.4 times lower than **1a**); however, the impact was less favorable than phosphate bridge elongation. Dinucleotide probes had generally weaker binding affinities than mononucleotide probes; the most potent dinucleotide probe **2d** had a *K*_D_ that was 1.3-fold higher than that of probe **1a**. In contrast, trinucleotide probes had a binding affinity in the range corresponding to mononucleotide probes. This result suggested that the third nucleotide eliminated the unfavorable influence of the second nucleotide by forming new contacts between eIF4E and the additional nucleotide or by rearrangement of the cap structure inside the eIF4E binding pocket. However, the *K*_D_ of oligonucleotide probe **4a** was sevenfold higher than that of **3a**, suggesting that further addition of nucleotides emulating the mRNA body may counteract this effect, resulting in negligible contribution of the mRNA body to the eIF4E:cap interaction.

**Table 1:** Binding affinities of mono-, di-, tri-, and oligonucleotide probes for eIF4E together with fluorescence enhancement factors (***g***).

L.p	*K*_D_ / nM	*g* = FL_bound_ / FL_free_
**1a**	59.7 ± 0.9	0.90 ± 0.02
**1b**	10.5 ± 1.0	0.98 ± 0.01
**1c**	20.7 ± 5.9	0.77 ± 0.02
**1e**	41.3 ± 4.4	1.10 ± 0.03
**1f**	63.7 ± 15.3	1.17 ± 0.07
**2a**	128.1 ± 11.0	1.10 ± 0.06
**2b**	108.6 ± 6.2	0.96 ± 0.04
**2c**	106.4 ± 7.2	0.91 ± 0.02
**2d**	79.8 ± 3.5	0.99 ± 0.05
**3a**	68.3 ± 14.4	0.56 ± 0.01
**3b**	33.9 ± 6.3	1.44 ± 0.04
**3c**	45.7 ± 7.4	1.15 ± 0.02
**4a**	477.4 ± 29.8	0.93 ± 0.02

FA experiments were performed in black 96-well plates using a Biotek Synergy H1 plate reader. Each well (200 µL) contained a fluorophore-tagged probe (0.5, 1, 2, or 10 nM) and increasing concentrations of the desired protein (from 0 to 2.5 µM).

For DcpS, we evaluated the hydrolysis-resistant probes **1d**, **1e**, **1f**, **2e**, **2f**, **2g**, **2h**, and **2i** (Table 2). Owing to the presence of a stereogenic P-center, the phosphorothioate probes existed in the form of two P-diastereoisomers, designated as D1 and D2 according to their order of elution during reverse-phase high-performance liquid chromatography. The diastereoisomers varied in binding affinity towards DcpS enzyme (e.g., probe **1f** had a *K*_D_ that was 1.8-fold lower than that of probe **1e**). Moreover, both phosphorothioate probes bound to DcpS with affinity higher than the corresponding probe with methylenobisphosphonate modification (1.3- and 2.4-times higher binding affinity compared with **1d**). Unexpectedly, probe **2d** carrying the 5′-phosphorothiolate moiety was found to be susceptible to DcpS-catalyzed hydrolysis under assay conditions and thus was not suitable for this assay, despite the fact that other compounds carrying this moiety have been shown to be resistant and potent inhibitors of DcpS^30^. The most promising probes for DcpS were found among dinucleotide cap analogs. The lowest *K*_D_ value was obtained for cap analog **2i** carrying a methylenebisphosphonate moiety and labeled at the 3′ position of ribose with a long linker.

**Table 2:** Binding affinities of mono- and dinucleotide probes for DcpS together with fluorescence enhancement factors (***g***).

L.p	Protein	*K*_D_ / nM	*g* = FL_bound_ / FL_free_
**1d**	DcpS	137 ± 5	0.91 ± 0.01
**1e**	DcpS	105 ± 12	0.88 ± 0.01
**1f**	DcpS	58 ± 2	0.80 ± 0.01
**2e**	DcpS	152 ± 32	0.93 ± 0.10
**2f**	DcpS	44 ± 5	0.71 ± 0.03
**2g**	DcpS	53 ± 13	1.88 ± 0.12
**2h**	DcpS	18 ± 6	1.54 ± 0.04
**2i**	DcpS	12.6 ± 0.9	1.12 ± 0.01

FA experiments were performed in black 96-well plates using a Biotek Synergy H1 plate reader. Each well (200 µL) contained a carboxyfluorescein-tagged probe (0.5, 1, 2, or 10 nM) and increasing concentrations of the desired protein (from 0 to 2.5 µM).

### Development and validation of an FA competitive binding assay for eIF4E

After preliminary evaluation of the probes, we aimed to develop an FA-based binding assay for eIF4E. To this end, we selected three mononucleotide probes characterized by medium to high binding affinity (**1a**, **1b**, and **1e**; Fig. 2A). Using these probes, we performed probe-displacement experiments (competitive FA assays), in which an unlabeled ligand competed with the fluorescent probe for protein binding (Fig. 2C–E). Thus, we selected eight known eIF4E binders, i.e., m^7^GMP, m^7^GDP, m^7^GTP, m^7^GpppG, m^7^Gpp_S_pG D1, m^7^Gpp_S_pG D2, m^7,2′-*O*^Gp_S_pppG D1, and m^7,2′-*O*^Gp_S_pppG D2, which showed a wide range of binding potencies. The binding curves obtained from FA competition assays were analyzed using four-parameter dose–response curves with a variable slope Hill equation (Fig. 2C–E)^45^. The determined EC_50_ and Hill slope values are shown in Table 3.

**Table 3:** Characterization of eight eIF4E ligands using three different probes in FA competition binding experiments.

Ligand	**Probe 1a**^a^		**Probe 1b**^b^		**Probe 1e**^c^	
	EC_50_ ± SD / nM	Hill slope	EC_50_ ± SD / nM	Hill slope	EC_50_ ± SD / nM	Hill slope
m^7^GMP	3608 ± 68	1.10 ± 0.10	5700 ± 1600	1.15 ± 0.29	4830 ± 450	0.96 ± 0.07
m^7^GDP	203.6 ± 5.5	1.36 ± 0.03	212 ± 30	1.16 ± 0.04	216 ± 11	1.08 ± 0.07
m^7^GTP	82.3 ± 8.5	1.89 ± 0.30	46 ± 10	1.16 ± 0.14	70.1 ± 8.5	1.51 ± 0.16
m^7^GpppG	413 ± 13	1.12 ± 0.03	599 ± 64	1.12 ± 0.06	551 ± 75	1.08 ± 0.11
m^7^Gpp_S_pG D1	184 ± 13	1.37 ± 0.02	210 ± 32	1.16 ± 0.18	226 ± 43	1.10 ± 0.06
m^7^Gpp_S_pG D2	382 ± 21	1.19 ± 0.06	511 ± 109	1.15 ± 0.06	477 ± 89	1.04 ± 0.17
m^7′-*O*^Gp_S_pppG D1	68 ± 3	2.07 ± 0.02	22.2 ± 3.7	1.64 ± 0.10	51.4 ± 6.1	1.46 ± 0.60
m^7′-*O*^Gp_S_pppG D2	74 ± 10	1.94 ± 0.26	34.9 ± 7.0	1.37 ± 0.08	64.3 ± 3.4	1.40 ± 0.06

The assay conditions were as follows: ^a^ 50 mM HEPES (pH 7.2) containing 100 mM KCl, 0.5 mM EDTA, and 1 mM DTT plus 10 nM probe **1a** and 100 nM eIF4E; ^b^ 50 mM HEPES (pH 7.2) containing 100 mM KCl, 0.5 mM EDTA, and 1 mM DTT plus 1 nM probe **1b** and 15 nM eIF4E; ^c^ 50 mM HEPES (pH 7.2) containing 100 mM KCl, 0.5 mM EDTA, and 1 mM DTT plus 1 nM probe **1c** and 100 nM eIF4E.

The results indicated that for low- and moderate-affinity ligands (half maximal inhibitory concentration [IC_50_] ≥ 200 nM), the Hill slope was close to 1, which was expected for a 1:1 binding model. However, the Hill slope was higher than 1 for high-affinity ligands, indicating that the probe affinity was too low to properly evaluate these ligands. As expected, the steepness of the curves was lowest for probe **1b**, which had the highest affinity for eIF4E. Hence, the results indicated that the high-affinity probe **1b** could be used to accurately measure the binding affinity of highly potent compounds, as also confirmed by the best correlation with the experimental data obtained from direct binding experiments using tsFQT (Fig. 2B).

**Figure 3:**
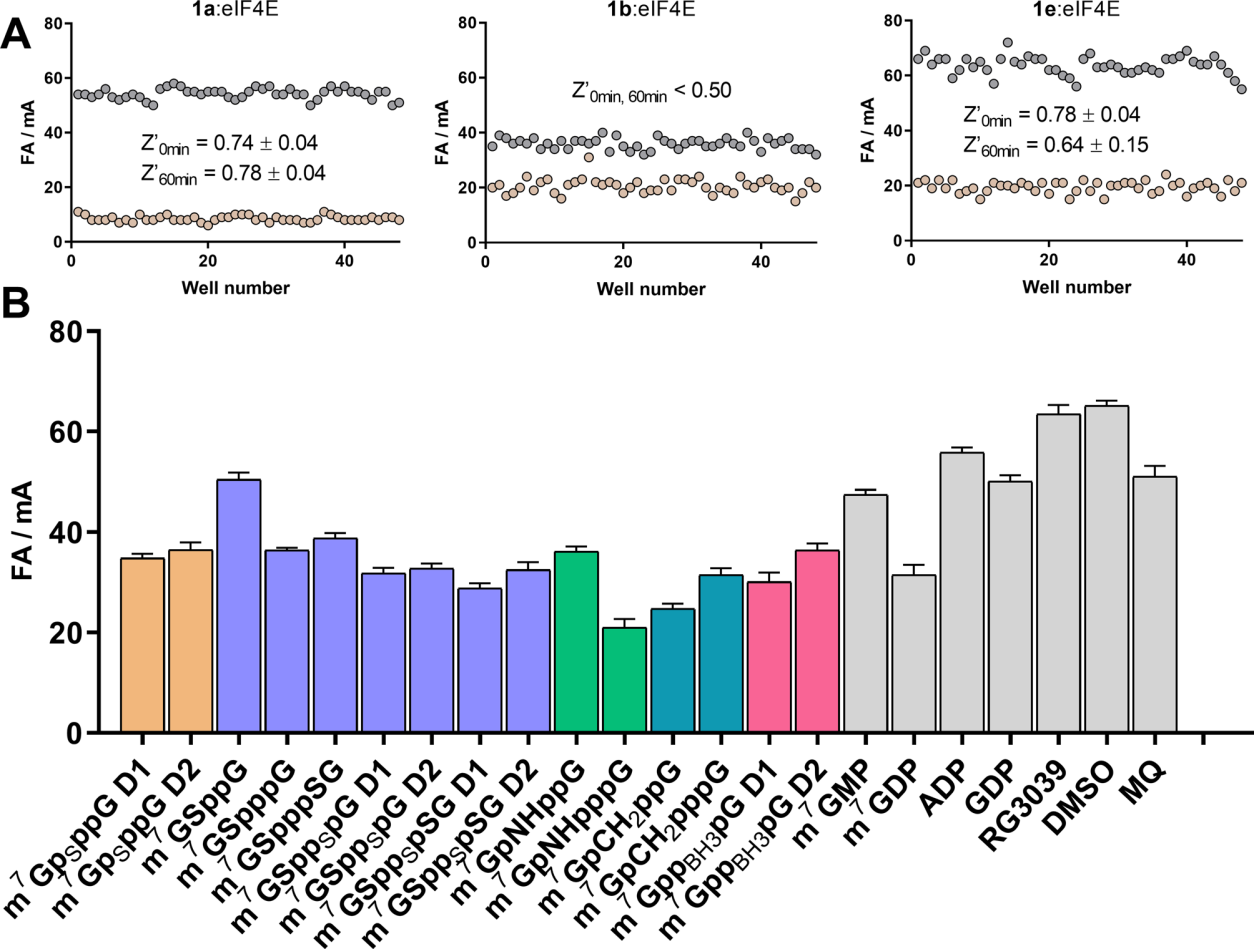
(**A**) Z’ factor measurements for eIF4E using three different probes (**1a**, **1b**, and **1e**). Assay conditions: 10 nM **1a**, 100 nM eIF4E, 1 µM m^7^GTP; 1 nM **1b**, 25 nM eIF4E, 1.5 µM m^7^GTP; 1 nM **1e**, 100 nM eIF4E, 1.5 µM m^7^GTP. (**B**) Screening experiment for eIF4E (1 nM **1e**, 100 nM eIF4E, 750 nM inhibitor).

Next, we evaluated whether FA assays based on probes **1a**, **1b**, or **1e** could be adopted for high-throughput screening. We first determined the assay quality based on Z’ factor estimation for all three systems, i.e., **1a:**eIF4E, **1b:**eIF4E, and **1e:**eIF4E (Fig. 3A). Probe-protein complex was used as a negative control sample (high FA), and a mixture of probe, eIF4E, and m^7^GTP (excess) was used as a positive control (low FA). The determined Z’ factors were 0.74 for the **1a**:eIF4E system and 0.78 for the **1e**:eIF4E system. After 60 min, the Z’ factors were still higher than 0.5. Thus, systems **1a:**eIF4E and **1e:**eIF4E could be successfully applied in a high-throughput screening format. Unfortunately, in a similar test for **1b**:eIF4E, we obtained a Z’ factor less than 0.5, with poor signal separation between positive and negative controls. Therefore, this system was considered inappropriate for high-throughput screening owing to the low signal-to-noise ratio. The reduced response window in comparison to other probes could be a result of increased rotational mobility caused by the additional phosphate group.

Using the **1e**:eIF4E system, a small in-house library of ligands was screened against eIF4E (Fig. 3B). The library contained mainly dinucleotide cap analogs modified within a triphosphate bridge, some standard mononucleotides, and non-nucleotide ligands. The binding affinities of these ligands were evaluated in previous studies. The screening was performed under optimized conditions in the presence of each tested inhibitor (750 nM). All tested dinucleotide cap analogs effectively competed for eIF4E, regardless of modification. However, the combination of an imidophosphate group with phosphate chain elongation appeared to have the most stabilizing effect on the protein–ligand complex. This observation was consistent with the literature data, showing that m^7^GpNHpppG had the highest association constant (*K*_AS_ = 112.3 ± 1.8 μM^−1^) among the tested ligands^46^. The screening also revealed the unfavorable impact of reducing the number phosphate groups on the binding (m^7^GSpppG to m^7^GSppG or m^7^GDP to m^7^GMP), consistent with literature data^30,47^. As expected, compounds without the m^7^G moiety did not bind to eIF4E. The allosteric inhibitor 4EGI-1, which binds to eIF4E at a different site than the cap, did not influence the fluorescence of the probe-protein complex under these conditions. All of the above results validated the FA method developed with probe **1e**.

### Testing of allosteric binding with eIF4E

eIF4E protein is a component of eukaryotic initiation translation complex 4F (eIF4F) and together with eIF4A and eIF4G proteins is required for initiation of the translation process^48^. 4EGI-1 is an inhibitor of eIF4E and eIF4G association and consequently leads to inhibition of cap-dependent translation^49^. Therefore, disruption of the eIF4E-eIF4G interaction is another important target for cancer therapy. For identification of small-molecule inhibitors of the eIF4E-eIF4G interaction, an FA assay has been developed previously^49^. The binding event was monitored by evaluating changes in FA resulting from the interaction of fluorescein-labeled 4G peptide with eIF4E with a *K*_D_ of 25 μM. Because only the 4G-binding site was observed, the potential connection between 4G- and cap-binding was not elucidated.

**Figure 4:**
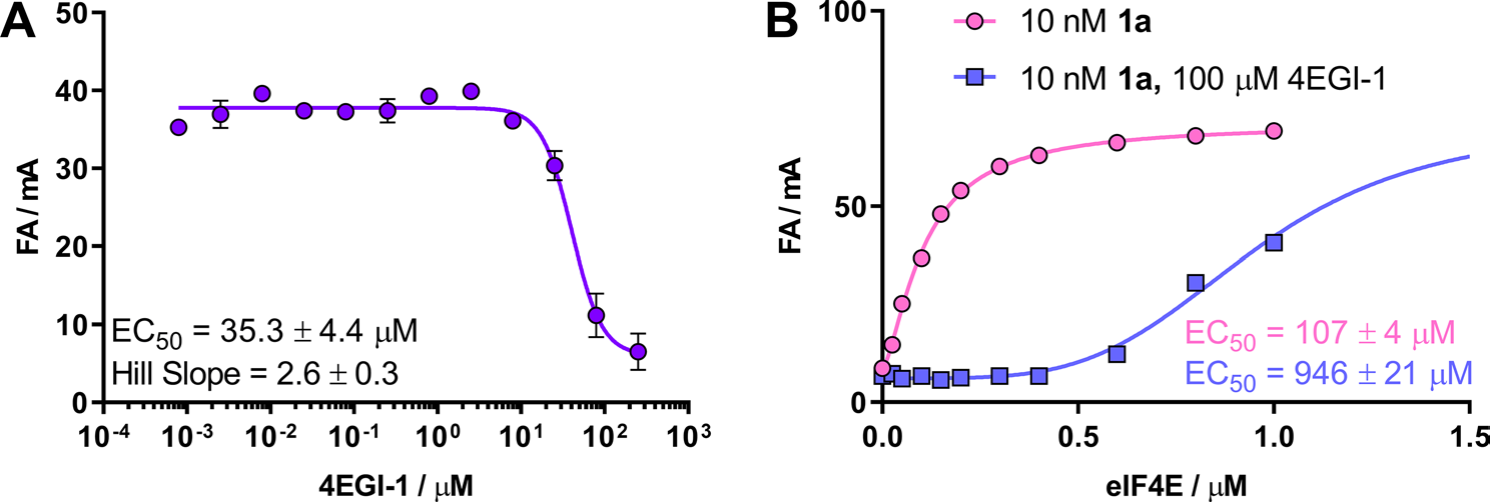
Testing of allosteric inhibition with probe **1a**. (**A**) Dose–response curves with the allosteric inhibitor 4EGI-1. Assay conditions: 10 nM **1a**, 100 nM eIF4E, and 50 mM HEPES buffer containing 100 mM KCl, 0.5 mM EDTA, and 1 mM DTT (pH 7.2). (**B**) Binding experiment in the absence/presence of 100 μM 4EGI-1. Assay conditions: 10 nM **1a** and 50 mM HEPES buffer containing 100 mM KCl, 0.5 mM EDTA, and 1 mM DTT (pH 7.2).

Next, we tested whether probe **1a** could be used to study the binding of inhibitors outside the cap-binding site, such as 4EGI-1. Although 4EGI-1 targets eIF4E at a binding site different from that of the fluorescent probes, we hypothesized that if 4EGI-1 binding evoked conformational changes in the proteins, FA readouts may be affected. Therefore, we conducted an experiment similar to the competitive test, but using increasing concentrations of 4EGI-1. Interestingly, we observed changes in the fluorescence anisotropy signal at 4EGI-1 concentrations exceeding 10 μM; the magnitude of these changes suggested that the fluorescent probe was released from the cap-binding site. The EC_50_ value for this interaction was 35.3 ± 4.4 μM (Fig. 4A). One possible explanation for this observation was that 4EGI-1 binding to eIF4E may trigger structural rearrangements, leading to allosteric inhibition of both interactions, i.e., cap-eIF4E and eIF4G-eIF4E^50^. To verify this, we performed direct binding assays for probe **1a** in the presence or absence of a high concentration of 4EGI-1 (100 μM; Fig. 4B). The results showed that the binding of probe **1a** to eIF4E was at least sevenfold weaker in the presence of 4EGI-1. This suggested the interdependence of the 4G- and cap-binding sites and revealed that our method could also be used for the identification and analysis of allosteric inhibitors of cap-dependent translation. For the first time, we showed that 4EGI-1 destabilized the cap-eIF4E complex.

### Capped oligonucleotide evaluation using FA

The biophysical aspects of cap-protein interactions are most often investigated using synthetically modified mono- and dinucleotide cap analogs. Despite many attempts to use fluorescently labeled and capped oligonucleotide probes to evaluate eIF4E binding^40^, their use is limited by synthetic complexity and consequently low availability. Therefore, we tested whether an FA-based competitive approach was suitable for evaluation of label-free capped oligonucleotides.

**Figure 5:**
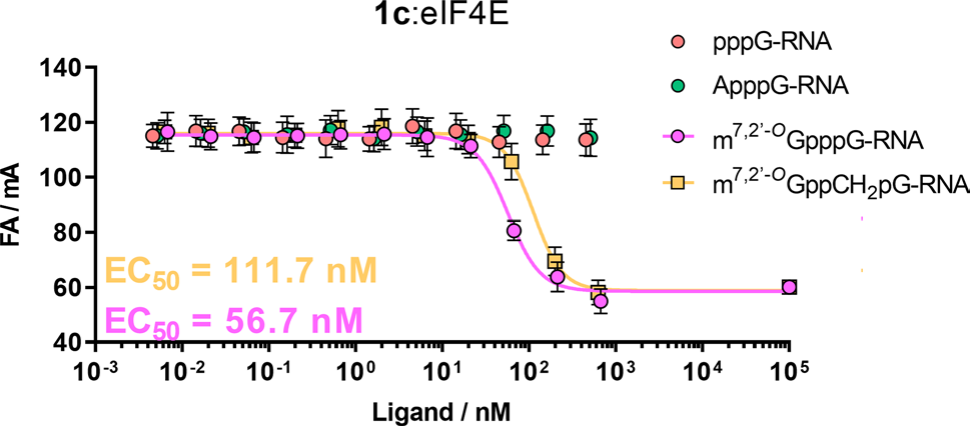
Dose–response curves for short (26 nt) RNAs with eIF4E. Assay conditions: systems 0.5 nM **1c** and 25 nM eIF4E were incubated with a serial half-log dilution of short RNAs at 25 °C. Fluorescence anisotropy was averaged over five measurements.

We tested whether our FA method could be applied to study short capped oligonucleotides. To this end, short 26-nt RNAs were prepared using in vitro transcription catalyzed by SP6 polymerase. Five different RNAs varying in the 5′ termini were prepared, i.e., m^7,2′-*O*^GpppG-RNA, m^7,2′-*O*^GppCH_2_pG-RNA, ApppG-RNA, and uncapped RNA (pppG-RNA). These RNAs were used as ligands in a competition experiment with probe **1c** to determine their EC_50_ values for eIF4E. As expected, we did not observe any binding event for pppG-RNA or ApppG-RNA (Fig. 5). In contract, m^7^G-capped oligonucleotides efficiently competed for interactions with eIF4E (EC_50_ values: 56.7 and 111.7 nM for m^7,2′-*O*^GpppG-RNA and m^7,2′-*O*^GppCH_2_pG-RNA, respectively). The obtained dose–response curves for the two capped RNAs and the determined EC_50_ values demonstrated the clear destabilizing effect of pCH_2_p modification on binding affinity to eIF4E (twofold lower EC_50_ for m^7,2′-*O*^GpppG-RNA compared with m^7,2′-*O*^GppCH_2_pG-RNA; Fig. 5). This observation was consistent with data obtained for 3′-ARCA dinucleotide cap analogs, in which the α/β-bisphosphonate modification weakens the affinity to eIF4E by approximately 2.3-times^38^. Thus, we showed that the FA method could be successfully used to study the affinity of oligonucleotides to eIF4E protein.

### Establishment and validation of an FA competitive assay for DcpS

**Figure 6:**
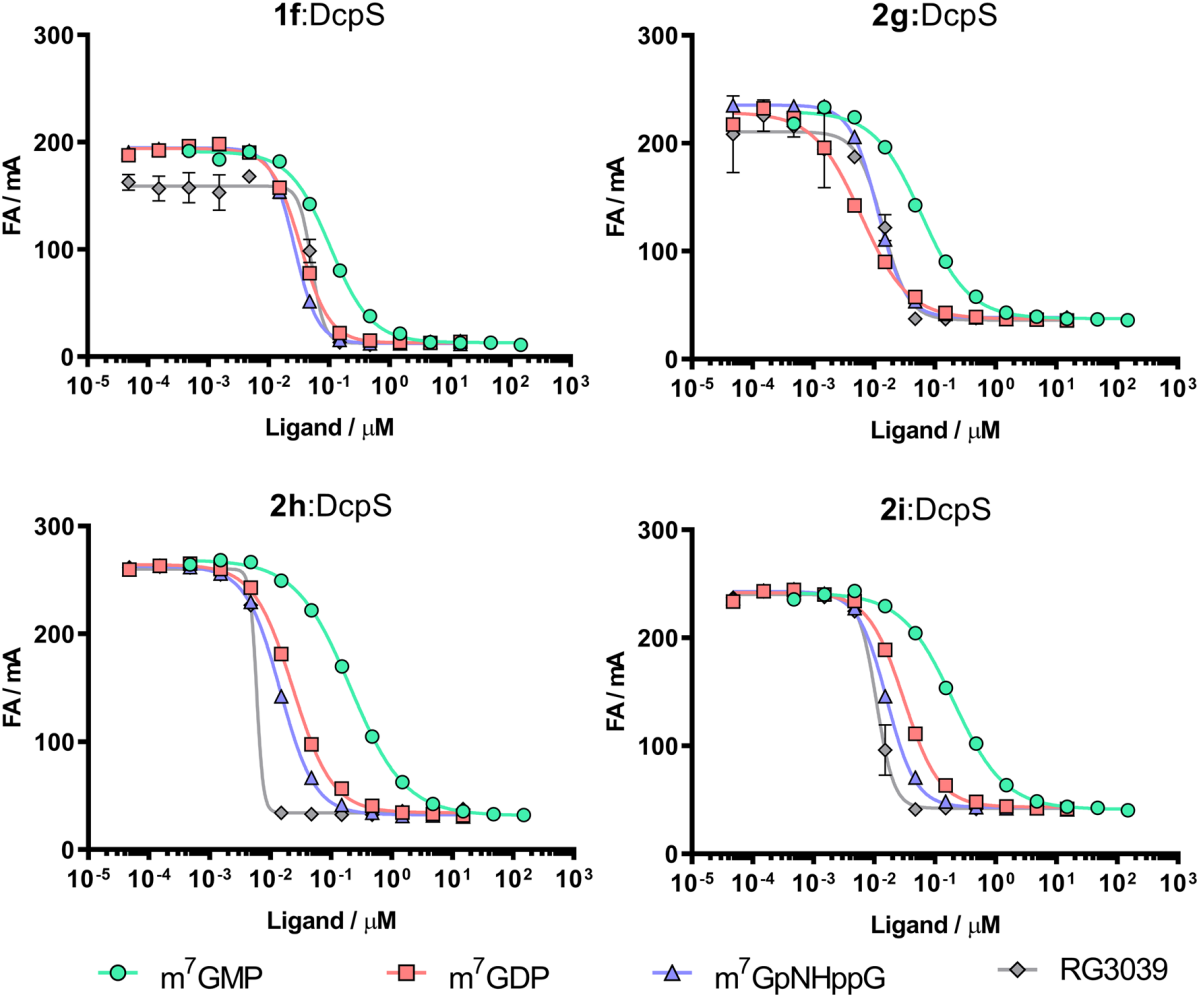
(**A**–**D**) Dose–response curves showing the inhibitory potencies of four inhibitors towards the DcpS enzyme using four different fluorescent probes. Systems: **1f** and 100 nM DcpS, 2 nM **2g** and 25 nM DcpS, 2 nM **2 h** and 25 nM DcpS, and 1 nM **2i** and 25 nM DcpS, each incubated with half-log serial dilutions of unlabeled ligand. Data shown are mean values ± standard deviations of at least two separated experiments, each performed in duplicate.

Using similar assumptions as the for eIF4E competition assay, we established conditions to study ligands of DcpS. For initial evaluation, we chose four high-affinity fluorescent probes, i.e., three dinucleotide probes (**2g**, **2 h**, and **2i**) and one mononucleotide probe (**1f**). Probes were tested with four DcpS inhibitors, i.e., m^7^GMP, m^7^GDP, m^7^GpNHppG, and RG3039, which differed in affinity to DcpS^33,46^. For each tested compound, we performed competition experiments to determine the EC_50_s of the selected probe:DcpS system (Fig. 6, Table 4). The affinities of the selected probes for DcpS increased in the following manner: **1f**  < **2g** < **2h** < **2i**. For the two lower affinity systems, i.e., **1f**:DcpS and **2g**:DcpS, we did not observe any separation of dose–response curves for three of the four inhibitors. In those systems, only the weak m^7^GMP inhibitor was accurately characterized. Characterization of the potent inhibitors was limited by insufficient probe affinity (Hill slope: 1.5–3.5). Using the high-affinity systems **2 h:DcpS** and **2i:DcpS**, all curves were sufficiently separated, even for the two most potent DcpS inhibitors (RG3039 and m^7^GpNHppG). The obtained dose–response curves for the highest affinity compound, i.e., RG3039, were characterized by high Hill slope values (> 3.6 for both systems). The binding curves obtained for RG3039 did not permit determination of affinity owing to the poor representation of binding curves because the total protein concentration significantly exceeded the *K*_D_ of the inhibitor. This result indicated that probes **2 h** and **2i** were still not optimal for quantitative studies of such potent inhibitors. Overall, we observed strong dependence of the ability to characterize potent inhibitors on the affinity of the probe (Fig. 6). Besides the limitations mentioned above, systems **2 h**:DcpS and **2i**:DcpS correctly assessed the inhibitory potencies of the selected inhibitors. The order of the tested compounds in terms of their binding affinities towards DcpS was consistent with data obtained using the fluoride-release (FR) fluorescent method^28^. FR assays use an artificial DcpS substrate, 7-methylguanosine 5′-fluoromonophosphate (m^7^GMPF), which is hydrolyzed by the enzyme to release fluoride. Fluoride activates the fluorogenic probe bis-(tert-butyldimethylsilylfluorescein) in a concentration-dependent manner; hence, the fluorescence signal is proportional to the enzymatic reaction progress. Using this activity-based assay, over 70 cap analogs were characterized as DcpS inhibitors, including compounds selected for FA method validation, i.e., m^7^GMP (IC_50_ = 97 ± 21 μM), m^7^GDP (IC_50_ = 5.2 ± 1.2 μM), m^7^GpNHppG (IC_50_ = 3.2 ± 0.9 μM), and RG3039 (IC_50_ = 0.048 ± 0.010 μM)^28^.

**Table 4:** EC_50_ and Hill slope parameters determined for selected inhibitors using **1f**:DcpS, **2g**:DcpS, **2h**:DcpS, and **2i**:DcpS systems.

Ligand	**1f** :DcpS		**2g**:DcpS		**2h**:DcpS		**2i**:DcpS	
	EC_50_ ± SD (nM)	Hill slope	EC_50_ ± SD (nM)	Hill slope	EC_50_ ± SD (nM)	Hill slope	EC_50_ ± SD (nM)	Hill slope
m^7^GMP	104 ± 11	1.19 ± 0.15	112 ± 11	1.04 ± 0.02	357 ± 44	0.93 ± 0.07	255 ± 14	1.00 ± 0.02
m^7^GDP	36.5 ± 3.6	1.98 ± 0.18	16.4 ± 0.2	1.55 ± 0.09	31.2 ± 10.7	1.06 ± 0.37	36.2 ± 4.7	1.42 ± 0.06
m^7^GpNHppG	27.8 ± 2.7	2.26 ± 0.16	11.6 ± 0.4	1.95 ± 0.07	18.1 ± 5.0	1.23 ± 0.44	17.9 ± 1.3	1.66 ± 0.10
RG3039	48.5 ± 3.7	3.03 ± 1.51	15.4 ± 2.7	3.52 ± 1.06	6.1 ± 0.6	7.66 ± 2.19	10.0 ± 2.0	3.67 ± 0.51

**Figure 7:**
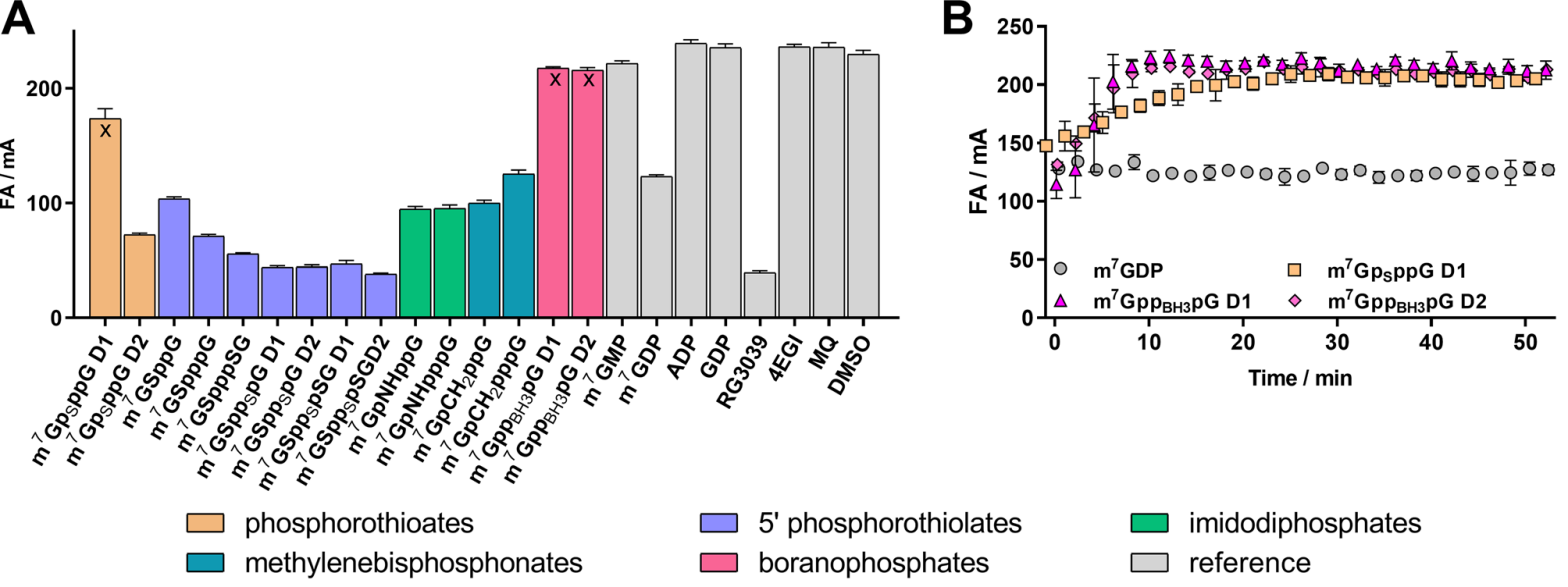
(**A**) Screening experiment for DcpS (1 nM **2i**, 25 nM DcpS, 1.5 µM inhibitor) using a small in-house library. (**B**) FA signal monitoring during the screening experiment for four selected compounds.

Because probe **2i** showed the lowest *K*_D_ value toward DcpS and was the most effective for characterization of potent inhibitors, such as RG3039, the **2i**:DcpS system was chosen for high-throughput method optimization. The Z’ factor was determined under conditions optimized for the competition assay. The Z’ value exceeded 0.8 for incubation times up to 1 h, making the assay suitable for screening experiments (Table 5). A screening experiment was then performed using the same compound library as that used for eIF4E screening. The results highlighted the impact of the triphosphate bridge modification on the affinity for the protein. Cap analogs modified with phosphorothioate and phosphorothiolate moieties (m^7^GSpp_S_pG D1, m^7^GSpp_S_pG D2, m^7^GSpp_S_pSG D1, and m^7^GSpp_S_pSG D2) were the most potent inhibitors. The combination of these two modifications afforded compounds with properties similar to RG3039, which was previously identified as a potent DcpS inhibitor using FR assays^30^. Cap analogs containing imidophosphate and methylenebisphosphonate moieties (e.g., m^7^GpCH_2_ppG, m^7^GpCH_2_pppG, m^7^GpNHppG, and m^7^GpNHpppG) were also strong DcpS inhibitors (showing an inhibitory potency similar to that of m^7^GDP) but were significantly weaker than RG3039. The FA method also enabled the identification of unstable compounds, e.g., hydrozylable ligands for which determination of affinity is problematic (as observed by FA signal changes during the experiment; Fig. 7). Among the tested ligands, m^7^Gp_S_ppG D1, m^7^Gpp_BH3_pG D1, and m^7^Gpp_BH3_pG D2 were recognized as slowly hydrolyzed DcpS substrates, which are difficult to identify using other screening methods. Despite the limitations of the FA method to characterize strong DcpS inhibitors, the screening assay was found to be suitable for the discovery and preliminary evaluation of DcpS inhibitors.

**Table 5:** Estimated Z’ factors for the **2i**:DcpS system.

Probe	Protein	Z’ factor (0 min) mean ± SD, n = 3	Z’ factor (60 min) mean ± SD, n = 3
2i	DcpS	0.83 ± 0.04	0.84 ± 0.01

Assay conditions: 1 nM **2i**, 25 nM DcpS, 1.5 µM m^7^GDP.

## Conclusions

FA is a powerful technique that is widely used to study protein–ligand interactions. In this study, we used FA to develop new methods for searching small-molecule inhibitors of cap-binding proteins. In the first step, we characterized a set of fluorescent probes. As probes, we used fluorescently labeled m^7^G nucleotide analogs resembling natural substrates or ligands interacting with test proteins. We verified the influence of the bridging modification and cap-fluorophore linker length on affinity towards eIF4E and DcpS and the tested fluorescence sensitivity to binding. Based on these studies, we selected the most promising probe candidates for competitive studies and ligand characterization. Selected probe:protein systems were used to determine EC_50_ and Hill slope parameters for known ligands of eIF4E and DcpS. The obtained values correlated well with literature data. Probes characterized by high affinity to the target and good FA responses were adapted to high-throughput screening assays.

As a result of this analysis, we developed FA methods for both eIF4E and DcpS. The methods could be successfully used for ligand screening purposes and EC_50_ parameter determination. eIF4E ligands have been extensively studied owing to the involvement of eIF4E in tumorigenesis and its role as a therapeutic target in many cancers. New ligands of eIF4E could facilitate the identification of novel anticancer agents. Notably, we found that the FA method could be used to study allosteric eIF4E ligands, such as 4EGI-1. Furthermore, high affinity of the 5′ cap for eIF4E is also crucial for the design of efficiently translated therapeutic mRNAs^51^. We showed that the FA method developed in this study was suitable for evaluation of small molecules as well as capped RNAs. This approach is a novel method that could be applied for the implementation of high-throughput approaches in therapeutic mRNA optimization and quality control. Screening of potential DcpS inhibitors is a new field of research, and few inhibitor families have been identified. DcpS plays general roles in the control of gene expression and has been independently linked to SMA, intellectual disability, and AML. Thus, identification of novel DcpS inhibitors could facilitate further studies of the connections between inhibitory and therapeutic effects because the mechanisms of action are still unknown.

## Methods

### Synthesis of fluorescent probes

Fluorescent probes **1a**–**1f**, **2a**–**2i** and **3a**–**3c** were synthesized chemically using methods based on phosphorimidazolide chemistry. The fluorescent labelling with fluorescein was carried out either by copper-catalyzed azide-alkyne cycloaddition or amide bond formation by N-hydroxysuccinimide chemistry. Further details on the chemical synthesis are included in the Supporting Information S1. Probe **4a** was purchased from TriLink Biotechnology.

### eIF4E and DcpS expression and purification

Murine eIF4E (residues 28–217) was expressed in E. coli and purified as described previously^26^. Briefly, high level expression of eIF4E obtained at conditions of 0.5 mM isopropyl-β-D-thiogalactoside (IPTG) at 37 °C induced the formation of inclusion bodies. Inclusion bodies containing eIF4E were solubilized in 50 mM HEPES/KOH (pH 7.2) buffer containing 10% glycerol, 6 M guanidine hydrochloride and 2 mM DTT. Protein was then refolded during a two-step dialysis against buffer with decreasing concentration of guanidine hydrochloride in the presence of 100 mM KCl. Subsequently eIF4E was loaded on ion exchange HiTrap SP HP column (GE Healthcare), eluted with linear gradient of 0.1–1 M KCl and finally desalted and polished during a gel filtration purification step on HiLoad 16/600 Superdex 75 pg (GE Healthcare) using 50 mM HEPES pH 7.2, 100 mM KCl, 0.5 mM EDTA, 2 mM DTT buffer. eIF4E was aliquoted and stored in the presence of 10% glycerol at −80 °C. Before each experiment the protein was centrifuged on Ultrafree-MC Centrifugal PVDF filter with 0.45 mm pore size (Millipore), at 4 °C and 3000×*g* for 2 min to remove any possible aggregates.

Expression of recombinant His-tagged human DcpS was performed in BL21(DE3) RIL strain and induced overnight at 18 °C using 0.5 mM IPTG as described previously^26^. Cells were harvested, resuspended in buffer (50 mM Tris pH 7.6, 500 mM NaCl, 20 mM imidazole) with lysozyme (0.1 mg/ml) and protease inhibitors (1 mM PMSF, 1 µM pepstatin A, 0.3 µM aprotinin) and then lysed using sonication. Lysate was clarified by centrifugation at 35 000 × g for 40 min at 4 °C. The cell supernatant was passed over a 5 mL HisTrap HP (GE Healthcare) affinity column and Ni–NTA-bound proteins were eluted using 50 mM Tris buffer pH 7.6 containing 500 mM NaCl and 400 mM imidazole. The enzyme hDcpS was purified to homogeneity on HiLoad 16/600 Superdex 200 pg (GE Healthcare) gel filtration column using 50 mM Tris·HCl pH 7.6, 200 mM NaCl, 2 mM DTT buffer. Protein was aliquoted and stored in the presence of 10% glycerol at -80 °C.

### Preparation of differently capped oligonucleotide ligands

Short RNAs were prepared as described previously^30^. RNAs were generated on template of annealed oligonucleotides (CAGTAATACGACTCACTATAGGGGAAGCGGGCATGCGGCCAGCCATAGCCGATCA and TGATCGGCTATGGCTGGCCGCATGCCCGCTTCCCCTATAGTGAGTCGTATTACTG), which contains T7 promoter sequence (TAATACGACTCACTATA) and encodes 35-nt long sequence (GGGGAAGCGGGCATGCGGCCAGCCATAGCCGATCA). Typical in vitro transcription reaction (100 µl) was incubated at 37 °C for 2 h and contained: RNA Pol buffer (40 mM Tris–HCl pH 7.9, 6 mM MgCl_2_, 1 mM DTT, 2 mM spermidine), 10 U/µl T7 RNA polymerase (ThermoFisher Scientific), 1 U/µl RiboLock RNase Inhibitor (ThermoFisher Scientific), 0.5 mM ATP/CTP/UTP, 0.125 mM GTP, 1.25 mM cap analog of interests and 0.1 µM annealed oligonucleotides as a template. Following 2 h incubation, 0.1 U/µl DNase I (ThermoFisher Scientific) was added and incubation was continued for 30 min at 37 °C. To generate uncapped RNA in reaction mixture cap analog was omitted whereas concentration of GTP was increased to 0.5 mM. The crude RNAs were purified using RNA Clean & Concentrator-25 (Zymo Research). Quality of transcripts was checked on 15% acrylamide/7 M urea gels, whereas concentration was determined spectrophotometrically. To remove in vitro transcription by-products of unintended size RNA samples were gel-purified using PAA elution buffer (0.3 M sodium acetate, 1 mM EDTA, 0.05% Triton X-100), precipitated with isopropanol and dissolved in water.

### FA binding assay

FA measurements were performed on a microplate reader Biotek Synergy H1 equipped with excitation (485 ± 20 nm) and emission (528 ± 20 nm) polarization filters. Experiments were carried out at 25 °C in 96-well non-binding microplates with sample volume of 200 µl per well. Two different buffers were used depending on protein used in the assay:

eIF4E assay buffer—50 mM HEPES (pH 7.2), 100 mM KCl, 0.5 mM EDTA, 1 mM DTT.

DcpS assay buffer—50 mM Tris–HCl (pH 7.6), 200 mM KCl, 0.5 mM EDTA, 1 mM DTT.

In the direct binding experiments aimed at determining *K*_d_ values for protein-probe complexes, the fluorescent probe at a constant concentration was mixed with an increasing concentration of the protein (0–2.5 μM). Concentration of fluorescent probes in binding experiment are provided in Table 6. Before FA measurements, the plates containing analyzed samples were incubated for 10 min at 25 °C with simultaneous shaking at 300 rpm, then the protein was added to each well, samples were incubated for additional 3 min. The FA readouts were performed in a microplate reader at 25 °C. FA signals were recorded for 20 min with 60 s interval.

**Table 6:** Protein and probe concentrations used in the FA binding assay.

	Protein	Probe	Probe concentration (nM)
1	eIF4E	**1a**	10
2	eIF4E	**1b**	1
3	eIF4E	**1c**	0.5
4	eIF4E	**1e**	1
5	eIF4E	**1f**	1
6	eIF4E	**2a**	10
7	eIF4E	**2b**	10
8	eIF4E	**2c**	10
9	eIF4E	**2d**	10
10	eIF4E	**2e**	10
11	eIF4E	**3a**	10
12	eIF4E	**3b**	1
13	eIF4E	**3c**	1
14	eIF4E	**4a**	10
15	DcpS	**1d**	10
16	DcpS	**1e**	10
17	DcpS	**1f**	10
18	DcpS	**2e**	10
19	DcpS	**2f.**	10
20	DcpS	**2g**	2
21	DcpS	**2h**	2
22	DcpS	**2i**	1

The FA values for each timepoint were calculated according to the following equation:

1 $$FA\left(\mathrm{mA}\right)= \frac{{I}_{\parallel }-G \bullet {I}_{\perp }}{{I}_{\parallel } + 2\bullet G \bullet {I}_{\perp }}\bullet 1000$$

where ${I}_{\parallel }$ is the parallel emission intensity, *P* is the perpendicular emission intensity, and *G* is the grating factor. The value of the G factor was equal 0.994.

For each sample, the final FA value taken to *K*_D_ determination was the mean FA value from all datapoints determined for timepoints between 10 and 20 min.

To determine the dissociation constants, FA values were plotted as a function of protein concentration and the binding curves were fitted using the following equation:

2 $$FA= {A}_{F}+({A}_{B}-{A}_{F})\frac{\left(c+ {L}_{T}+{K}_{D}\right)-\sqrt{{(c+{L}_{T}+{K}_{D })}^{2}-4\bullet c\bullet {L}_{T}}}{2 {L}_{T}}$$

where *FA* is the determined fluorescence anisotropy, *A*_*F*_ is the fluorescence anisotropy of free probe, *A*_*B*_ is the fluorescence anisotropy of probe-protein complex, *L*_*T*_ is the total ligand concentration.

The following equation was used to calculate total fluorescence intensity of the probe:

3 $${I}_{T}= {I}_{\parallel }+2{I}_{\perp }$$

where ${I}_{T}$—total fluorescence intensity, ${I}_{\parallel }$—the parallel emission intensity, ${I}_{\perp }$—the perpendicular emission intensity.

The calculated values of the total fluorescence intensity were plotted against the protein concentration and the curve described by the Eq. 2 was fitted. Enhancement factor *g* was determined from the following equation:

4 $$g= \frac{{A}_{bound}}{{A}_{free}}\bullet 100\%$$

If the change in total fluorescence intensity due to binding was greater than 10%, the correction for the calculation of the bound fraction of probe was applied^44^:

5 $${f}_{b}= \frac{A-{A}_{free}}{{A}_{bound }-{A}_{free}+(g-1)({A}_{bound}-A)}$$

where $A$—measured fluorescence anisotropy, ${A}_{free}$—the fluorescence anisotropy of free probe, ${A}_{bound}$—the fluorescence anisotropy of probe-protein complex, $g$—enhancement factor.

### FA competition assay

For competitive binding assay, a mixture containing the probe and the protein was incubated with the tested ligand. The exact concentrations of eIF4E or DcpS used for evaluation of particular probes are summarized in Table 7.

**Table 7:** Protein and probe concentrations used in the competitive binding assays.

	Protein	Protein concentration (nM)	Probe	Probe concentration (nM)
1^a^	eIF4E	100	**1a**	10
2	eIF4E	15	**1b**	1
3^b^	eIF4E	50	**1c**	0.5
4	eIF4E	100	**1e**	1
5	eIF4E	100	**1f**	1
6	DcpS	25	**2g**	2
7	DcpS	25	**2h**	2
8	DcpS	25	**2i**	1

^a^System used to characterize 4EGI-1.

^b^System used for experiments with oligonucleotides.

In competitive measurements, a constant concentration of the protein and fluorescent probe and increasing concentration of the test ligand were used. At least 12 point dilutions of the tested compound were used. The experiments were carried out in 96-well plates. Each sample contained a mixture consisting of the probe, tested ligand and buffer (same as for the direct binding assay). The samples were incubated for 10 min at 25 °C with simultaneous shaking, then the protein was added to each well, incubated for additional 3 min, followed by the measurement of fluorescence anisotropy. FA signals were recorded for 20 min with 2 min intervals. For each sample, the final FA value taken to *EC*_50_ determination was the mean FA value from all datapoints determined for timepoints between 10 and 20 min.

The *EC*_50_ value, *i.e.*, the ligand concentration required for 50% displacement of the probe from the complex with protein were calculated according to the equation:

6 $$FA=Bottom+\frac{\left(Top-Bottom\right)}{1+ \frac{{EC}_{50}^{HillSlope}}{{{L}}^{HillSlope}}}$$

where *FA* is the measured fluorescence anisotropy, *Top* and *Bottom* are asymptotes, *L* is the ligand concentration, *HillSlope* is the steepness of the curve.

### Quality assessment and screening of an in-house compound library

To evaluate the quality of the assay, the Z’ factor was determined for several protein-probe combinations. The assay was performed on 96-well microplates, where half of the samples were negative controls and the other half were positive controls. Negative control samples contained fluorescent probe and the tested protein, while the positive control contained additionally saturating concentration of high affinity ligand (m^7^GTP for eIF4E and m^7^GDP for DcpS). The concentration used in each experiment were as follows: 10 nM **1a**, 100 nM eIF4E, (1 µM m^7^GTP); 1 nM **1b**, 25 nM eIF4E, (1.5 µM m^7^GTP); 1 nM **1e**, 100 nM eIF4E, (1.5 µM m^7^GTP); 1 nM **2i**, 25 nM DcpS, (1.5 µM m^7^GDP).

The Z’ factor values were calculated according to the following equation:

7 $${Z}^{^{\prime}}=1- \frac{{3SD}_{n}+{3SD}_{p}}{{\mu }_{n}- {\mu }_{p}}$$

where *SD*_*n*_ and *SD*_*p*_ are the standard deviations, and *μ*_*n*_ and *μ*_*p*_ represent the means of the FA values obtained from the negative and positive controls, respectively.

For the screening experiments a small in-house library containing 21 ligands was used. Experiments were conducted in the same manner as Z’ factor determination, with exception that the ligand concentration was modified (eIF4E screening conditions: 1 nM **1e**, 100 nM eIF4E, 750 nM ligand, DcpS screening conditions: 1 nM **2i**, 25 nM DcpS, 1.5 µM ligand). For eIF4E screening experiment, the **1e**:eIF4E system was used, for DcpS screening **2i**:DcpS system was used.

## Supplementary Information

Supplementary information.

## References

[1] Furuichi Y, Muthukrishnan S, Shatkin AJ (1975). 5'-Terminal m-7G(5')ppp(5')G-m-p in vivo: identification in reovirus genome RNA. Proc. Natl. Acad. Sci. USA.

[2] Quiocho FA, Hu G, Gershon PD (2000). Structural basis of mRNA cap recognition by proteins. Curr. Opin. Struct. Biol..

[3] Niedzwiecka A, Marcotrigiano J, Stepinski J, Jankowska-Anyszka M, Wyslouch-Cieszynska A, Dadlez M, Gingras AC, Mak P, Darzynkiewicz E, Sonenberg N, Burley SK, Stolarski R (2002). Biophysical studies of eIF4E cap-binding protein: recognition of mRNA 5' cap structure and synthetic fragments of eIF4G and 4E-BP1 proteins. J. Mol. Biol..

[4] Topisirovic I, Svitkin YV, Sonenberg N, Shatkin AJ (2011). Cap and cap-binding proteins in the control of gene expression. Wiley Interdiscip. Rev. RNA.

[5] Gu M, Lima CD (2005). Processing the message: structural insights into capping and decapping mRNA. Curr. Opin. Struct. Biol..

[6] Lewis JD, Izaurralde E (1997). The role of the cap structure in RNA processing and nuclear export. Eur. J. Biochem..

[7] Rau M, Ohlmann T, Morley SJ, Pain VM (1996). A reevaluation of the cap-binding protein, eIF4E, as a rate-limiting factor for initiation of translation in reticulocyte lysate. J. Biol. Chem..

[8] Raught B, Gingras AC (1999). eIF4E activity is regulated at multiple levels. Int. J. Biochem. Cell Biol..

[9] Lazaris-Karatzas A, Montine KS, Sonenberg N (1990). Malignant transformation by a eukaryotic initiation factor subunit that binds to mRNA 5' cap. Nature.

[10] De Benedetti A, Harris AL (1999). eIF4E expression in tumors: its possible role in progression of malignancies. Int. J. Biochem. Cell Biol..

[11] Truitt ML, Conn CS, Shi Z, Pang X, Tokuyasu T, Coady AM, Seo Y, Barna M, Ruggero D (2015). Differential requirements for eIF4E dose in normal development and cancer. Cell.

[12] Chen X, Kopecky DJ, Mihalic J, Jeffries S, Min X, Heath J, Deignan J, Lai S, Fu Z, Guimaraes C, Shen S, Li S, Johnstone S, Thibault S, Xu H, Cardozo M, Shen W, Walker N, Kayser F, Wang Z (2012). Structure-guided design, synthesis, and evaluation of guanine-derived inhibitors of the eIF4E mRNA-cap interaction. J. Med. Chem..

[13] Carroll M, Borden KL (2013). The oncogene eIF4E: using biochemical insights to target cancer. J. Interferon Cytokine Res..

[14] Bail S, Kiledjian M (2008). DcpS, a general modulator of cap-binding protein-dependent processes?. RNA Biol..

[15] Singh J, Salcius M, Liu SW, Staker BL, Mishra R, Thurmond J, Michaud G, Mattoon DR, Printen J, Christensen J, Bjornsson JM, Pollok BA, Kiledjian M, Stewart L, Jarecki J, Gurney ME (2008). DcpS as a therapeutic target for spinal muscular atrophy. ACS Chem. Biol..

[16] Ng CK, Shboul M, Taverniti V, Bonnard C, Lee H, Eskin A, Nelson SF, Al-Raqad M, Altawalbeh S, Séraphin B, Reversade B (2015). Loss of the scavenger mRNA decapping enzyme DCPS causes syndromic intellectual disability with neuromuscular defects. Hum. Mol. Genet..

[17] Yamauchi T, Masuda T, Canver MC, Seiler M, Semba Y, Shboul M, Al-Raqad M, Maeda M, Schoonenberg VAC, Cole MA, Macias-Trevino C, Ishikawa Y, Yao Q, Nakano M, Arai F, Orkin SH, Reversade B, Buonamici S, Pinello L, Akashi K, Bauer DE, Maeda T (2018). Genome-wide CRISPR-Cas9 screen identifies Leukemia-specific dependence on a pre-mRNA metabolic pathway regulated by DCPS. Cancer Cell.

[18] Meziane O, Piquet S, Bossé GD, Gagné D, Paquet E, Robert C, Tones MA, Simard MJ (2015). The human decapping scavenger enzyme DcpS modulates microRNA turnover. Sci. Rep..

[19] Howell MD, Singh NN, Singh RN (2014). Advances in therapeutic development for spinal muscular atrophy. Fut. Med. Chem..

[20] Gogliotti RG, Cardona H, Singh J, Bail S, Emery C, Kuntz N, Jorgensen M, Durens M, Xia B, Barlow C, Heier CR, Plasterer HL, Jacques V, Kiledjian M, Jarecki J, Rusche J, DiDonato CJ (2013). The DcpS inhibitor RG3039 improves survival, function and motor unit pathologies in two SMA mouse models. Hum. Mol. Genet..

[21] Alesi V, Capolino R, Genovesea S, Capriati T, Loddo S, Calvieri G, Calacci C, Diociaiuti A, Diamanti A, Novelli A, Dallapiccola B (2018). An additional patient with a homozygous mutation in DCPS contributes to the delination of Al-Raqad syndrome. Am. J. Med. Genet. A.

[22] Ahmed I, Buchert R, Zhou M, Jiao X, Mittal K, Sheikh TI, Scheller U, Vasli N, Rafiq MA, Brohi MQ, Mikhailov A, Ayaz M, Bhatti A, Sticht H, Nasr T, Carter MT, Uebe S, Reis A, Ayub M, John P, Kiledjian M, Vincent JB, Jamra RA (2015). Mutations in DCPS and EDC3 in autosomal recessive intellectual disability indicate a crucial role for mRNA decapping in neurodevelopment. Hum. Mol. Genet..

[23] Moerke NJ (2009). Fluorescence polarization (FP) assays for monitoring peptide-protein or nucleic acid-protein binding. Curr. Protoc. Chem. Biol..

[24] Visco C, Perrera C, Thieffine S, Sirtori FR, D'Alessio R, Magnaghi P (2012). Development of biochemical assays for the identification of eIF4E-specific inhibitors. J. Biomol. Screen.

[25] Natarajan A, Moerke N, Fan YH, Chen H, Christ WJ, Wagner G, Halperin JA (2004). Synthesis of fluorescein labeled 7-methylguanosinemonophosphate. Bioorg. Med. Chem. Lett..

[26] Kasprzyk R, Starek BJ, Ciechanowicz S, Kubacka D, Kowalska J, Jemielity J (2019). Fluorescent turn-on probes for the development of binding and hydrolytic activity assays for mRNA cap-recognizing proteins. Chemistry.

[27] Kasprzyk R, Kowalska J, Wieczorek Z, Szabelski M, Stolarski R, Jemielity J (2016). Acetylpyrene-labelled 7-methylguanine nucleotides: unusual fluorescence properties and application to decapping scavenger activity monitoring. Org. Biomol. Chem..

[28] Baranowski MR, Nowicka A, Jemielity J, Kowalska J (2016). A fluorescent HTS assay for phosphohydrolases based on nucleoside 5'-fluorophosphates: its application in screening for inhibitors of mRNA decapping scavenger and PDE-I. Org. Biomol. Chem..

[29] Wanat P, Kasprzyk R, Kopcial M, Sikorski PJ, Strzelecka D, Jemielity J, Kowalska J (2018). ExciTides: NTP-derived probes for monitoring pyrophosphatase activity based on excimer-to-monomer transitions. Chem. Commun. (Camb).

[30] Wojtczak BA, Sikorski PJ, Fac-Dabrowska K, Nowicka A, Warminski M, Kubacka D, Nowak E, Nowotny M, Kowalska J, Jemielity J (2018). 5'-Phosphorothiolate dinucleotide cap analogues: reagents for messenger RNA modification and potent small-molecular inhibitors of decapping enzymes. J. Am. Chem. Soc..

[31] Golojuch S, Kopcial M, Strzelecka D, Kasprzyk R, Baran N, Sikorski PJ, Kowalska J, Jemielity J (2020). Exploring tryptamine conjugates as pronucleotides of phosphate-modified 7-methylguanine nucleotides targeting cap-dependent translation. Bioorg. Med. Chem..

[32] Walczak S, Nowicka A, Kubacka D, Fac K, Wanat P, Mroczek S, Kowalska J, Jemielity J (2017). A novel route for preparing 5' cap mimics and capped RNAs: phosphate-modified cap analogues obtained. Chem. Sci..

[33] Wypijewska A, Bojarska E, Lukaszewicz M, Stepinski J, Jemielity J, Davis RE, Darzynkiewicz E (2012). 7-methylguanosine diphosphate (m(7)GDP) is not hydrolyzed but strongly bound by decapping scavenger (DcpS) enzymes and potently inhibits their activity. Biochemistry.

[34] Kopcial, M., Wojtczak, B. A., Kasprzyk, R., Kowalska, J., Jemielity, J., N1-Propargylguanosine Modified mRNA Cap Analogs: Synthesis, reactivity, and applications to the study of cap-binding proteins. *Molecules* **2019,** *24* (10). 10.3390/molecules24101899 PMC6572376 31108861

[35] Piecyk K, Darzynkiewicz ZM, Jankowska-Anyszka M, Ferenc-Mrozek A, Stepinski J, Darzynkiewicz E, Bojarska E (2015). Effect of different N7 substitution of dinucleotide cap analogs on the hydrolytic susceptibility towards scavenger decapping enzymes (DcpS). Biochem. Biophys. Res. Commun..

[36] Lakowicz JR (1999). Principles of Fluorescence Spectroscopy.

[37] Hall MD, Yasgar A, Peryea T, Braisted JC, Jadhav A, Simeonov A, Coussens NP (2016). Fluorescence polarization assays in high-throughput screening and drug discovery: a review. Methods Appl. Fluoresc..

[38] Rydzik AM, Lukaszewicz M, Zuberek J, Kowalska J, Darzynkiewicz ZM, Darzynkiewicz E, Jemielity J (2009). Synthetic dinucleotide mRNA cap analogs with tetraphosphate 5',5' bridge containing methylenebis(phosphonate) modification. Org. Biomol. Chem..

[39] Kowalska J, Lukaszewicz M, Zuberek J, Ziemniak M, Darzynkiewicz E, Jemielity J (2009). Phosphorothioate analogs of m7GTP are enzymatically stable inhibitors of cap-dependent translation. Bioorg. Med. Chem. Lett..

[40] Fuchs AL, Neu A, Sprangers R (2016). A general method for rapid and cost-efficient large-scale production of 5' capped RNA. RNA.

[41] Dandliker WB, Hsu ML, Levin J, Rao BR (1981). Equilibrium and kinetic inhibition assays based upon fluorescence polarization. Methods Enzymol..

[42] Lundblad JR, Laurance M, Goodman RH (1996). Fluorescence polarization analysis of protein-DNA and protein-protein interactions. Mol. Endocrinol..

[43] Ozers MS, Hill JJ, Ervin K, Wood JR, Nardulli AM, Royer CA, Gorski J (1997). Equilibrium binding of estrogen receptor with DNA using fluorescence anisotropy. J. Biol. Chem..

[44] Zhang H, Wu Q, Berezin MY (2015). Fluorescence anisotropy (polarization): from drug screening to precision medicine. Expert Opin. Drug Discov..

[45] Gadagkar SR, Call GB (2015). Computational tools for fitting the Hill equation to dose-response curves. J. Pharmacol. Toxicol. Methods.

[46] Rydzik AM, Kulis M, Lukaszewicz M, Kowalska J, Zuberek J, Darzynkiewicz ZM, Darzynkiewicz E, Jemielity J (2012). Synthesis and properties of mRNA cap analogs containing imidodiphosphate moiety–fairly mimicking natural cap structure, yet resistant to enzymatic hydrolysis. Bioorg. Med. Chem..

[47] Wypijewska del Nogal A, Surleac MD, Kowalska J, Lukaszewicz M, Jemielity J, Bisaillon M, Darzynkiewicz E, Milac AL, Bojarska E (2013). Analysis of decapping scavenger cap complex using modified cap analogs reveals molecular determinants for efficient cap binding. FEBS J.

[48] Gingras AC, Raught B, Sonenberg N (1999). eIF4 initiation factors: effectors of mRNA recruitment to ribosomes and regulators of translation. Annu. Rev. Biochem..

[49] Moerke NJ, Aktas H, Chen H, Cantel S, Reibarkh MY, Fahmy A, Gross JD, Degterev A, Yuan J, Chorev M, Halperin JA, Wagner G (2007). Small-molecule inhibition of the interaction between the translation initiation factors eIF4E and eIF4G. Cell.

[50] Salvi N, Papadopoulos E, Blackledge M, Wagner G (2016). The Role of Dynamics and Allostery in the Inhibition of the eIF4E/eIF4G Translation Initiation Factor Complex. Angew Chem. Int. Ed. Engl..

[51] Hajj, K. A., Whitehead, K. A., Tools for translation: non-viral materials for therapeutic mRNA delivery. *Nature Reviews Material* **2017,** *2* (17056).

