# Supplementary material for: Evaluation of carboxyfluorescein-labeled 7-methylguanine nucleotides as probes for studying cap-binding proteins by fluorescence anisotropy
Source: Sci Rep. 2021 Apr 8;11:7687. doi: 10.1038/s41598-021-87306-8 (PMC8032668; doi:10.1038/s41598-021-87306-8)
Supplement: Supplementary file 1 — Supplementary information. [file 41598_2021_87306_MOESM1_ESM.docx]

Evaluation of carboxyfluorescein-labeled 7-methylguanine nucleotides as probes for studying cap-binding proteins by fluorescence anisotropy

Anna Wojtczak^a^, Renata Kasprzyk^b,c^, Marcin Warmiński^a^, Krystian Ubych^a,c^, Dorota Kubacka^a^, Pawel J. Sikorski^b^, Jacek Jemielity^b,^*, Joanna Kowalska^a,^*

^a^Division of Biophysics, Institute of Experimental Physics, Faculty of Physics, University of Warsaw, Ludwika Pasteura 5, 02-093 Warsaw, Poland

^b^Centre of New Technologies, University of Warsaw, Stefana Banacha 2c, 02-097 Warsaw, Poland

^c^College of Inter-Faculty Individual Studies in Mathematics and Natural Sciences, University of Warsaw, Stefana Banacha 2c, 02-097 Warsaw, Poland

*corresponding authors: [j.jemielity@cent.uw.edu.pl](mailto:j.jemielity@cent.uw.edu.pl), jkowalska@fuw.edu.pl

**Supplementary information**


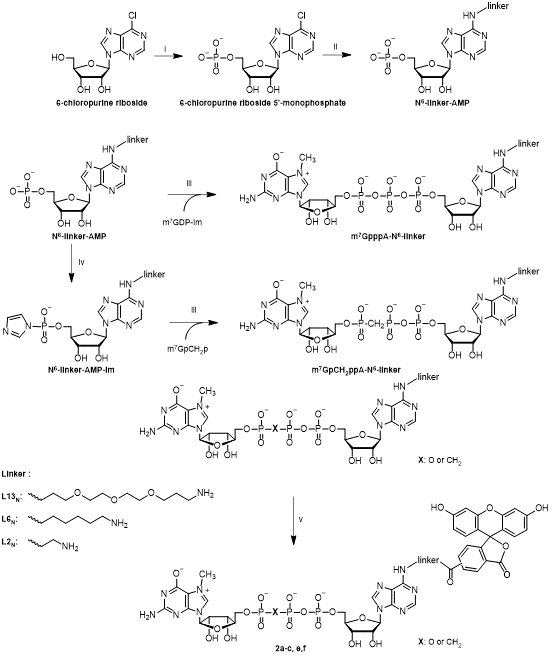


**Figure S1.** Synthetic pathway to *N*^6^-adenine modified compounds (**2a-c, e, f)**. i: POCl_3_, (CH_3_O)_3_PO (79%); ii: diamine linker, H_2_O, RT (92 - 98%); iii: (1) ZnCl_2_, DMSO; (2) EDTA, H_2_O, NaHCO_3_ (45–92%); iv: (1) imidazole, 2,2'-dithiodipyridine, Ph_3_P, DMF; (2) NaClO_4_, acetonitrile (cold) (85%); v: 5(6)-carboxyfluorescein, TEA, TSTU, DMSO, borate buffer pH 8.5 (12–50%)


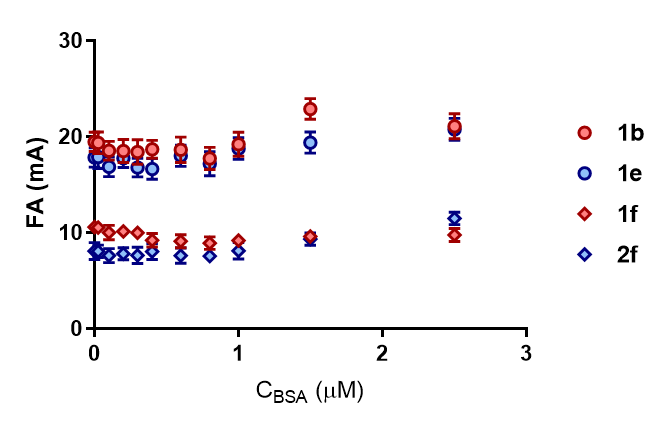


**Figure S2.** Fluorescence anisotropy for select probes as a function of Bovine Serum Albumin (BSA) concentration.

**Chemical syntheses**

**General information**

*Purification.* The synthesised nucleotides were purified by ion-exchange chromatography on a DEAE Sephadex A-25 (HCO_3_^-^ form) column. For this, the column was loaded with the reaction mixture and washed thoroughly with water until the eluate did not precipitate with AgNO_3_ solution, in order to remove solvents and unbound reagents. Nucleotides were then eluted using a linear gradient of triethylammonium hydrogen carbonate (TEAB) in deionised water. Collected fractions were analysed spectrophotometrically at 260 nm. Those containing the desired compound were combined and the purity was determined by RP HPLC. The yields were calculated on the basis of optical density miliunits (mOD = absorbance of the solution multiplied by its volume in mL) of the isolated products and corresponding starting materials (nucleotides or nucleotide *P*-imidazolide derivatives). Absorbance measurements were performed in phosphate buffer (pH 7.0, 0.1 M) at 260 nm for all nucleotides, except for m^7^G mononucleotide derivatives, for which phosphate buffer (pH 6.0, 0.1 M) was used. The pooled fractions were concentrated under reduced pressure with repeated additions of ethanol (96 %) and then acetonitrile (to decompose TEAB and to remove residual water, respectively) and the nucleotides were isolated as triethylammonium salts. The final compounds were purified by RP HPLC on a semi-preparative column (Discovery RP Amide C-16 250x21.2 mm, 5 μm or Grace Vision HT C18 HL 250x22 mm, 10 μm, flow rate 5.0 mL min^-1^) or an analytical column (Supelcosil LC-18-T 250x4.6 mm, 5 μm, flow rate 1.3 mL min^-1^) with UV detection at 254 nm. After repeated freeze-drying of the collected fractions the products were isolated as ammonium salts.

**Synthesis of fluorescent probes and their precursors**

*Starting materials.* Commercially available GMP disodium salt was converted into triethylammonium salt before synthesis by passing an aqueous solution of GMP disodium salt through Dowex 50W-X8 cationite resin in the triethylammonium form. The collected eluate was concentrated under reduced pressure with repeated additions of ethanol, and the residue was dried in a vacuum over P_4_O_10_ to yield the triethylammonium salt as a white solid.

Following compounds were synthesized according to the previously published protocols:

GpCH_2_p, m^7^GpCH_2_p, m^7^GDP, m^7^GDP-Im were synthesized as described previously, with minor modifications.^1^ N^6^-L2_N_-AMP, N^6^-L6_N_-AMP, N^6^‑L13_N_-AMP were synthesized as described previously.^2^ Alkyne-modified mononucleotide analogs (m^7^Gp_4_C_4_H_5_, m^7^Gp_5_C_4_H_5_, m^7^GpCH_2_ppC_4_H_5_, m^7^GTP_α_SOC_3_H_3_ D1, m^7^GTP_α_SOC_3_H_3_ D2), probe **1a**, 5-*N*-(2-azidoethyl)-fluorescein amide [(5)FAM-azide] and 6-*N*-(2-azidoethyl)-fluorescein amide [(6)FAM-azide] were synthesized as described previously.^3-5^ L6_N_-m^7^GDP and m^7^GpCH_2_ppG-L13_N_ were synthesized as described earlier.^6^ m^7^GpCH_2_ppG-L3_Y_ and m^7^G^5′S^pppG_m_-L3_Y_ were synthesized according to the previously published protocols, with minor modifications. Dinucleotide 5′‑phosphates pApG and pA_m_pG were synthesized by phosphoramidite method on a solid support and isolated as triethylammonium salts by ion-exchange chromatography on DEAE Sephadex as described previously.^7^ Probe **4a** was purchased from TriLink Biotechnology.

**Synthesis of 1b**

Aqueous solution of m^7^Gp_4_C_4_H_5_ triethylammonium salt (4.9 mg, 4.5 μmol, 28 μL, 0.16 M) was mixed with a solution of 6-FAM-azide (4.1 mg, 9.2 μmol) in DMSO (28 μL), followed by addition of 1.0 M aqueous solutions of CuSO_4_∙5H_2_O (1.2 mg, 4.8 μmol, 4.8 μL) and 1.0 M sodium ascorbate (1.8 mg, 9.1 μmol, 9.1 μL). The mixture was vortexed at a room temperature for 3 h and then the reaction was quenched by addition of Na_2_EDTA (1.8 mg, 4.8 μmol) and water (300 μL). The precipitated fluorescein dye was separated from the mixture by centrifugation. The reaction product was purified by analytical RP HPLC to give **1b** (0.64 mg, 0.55 μmol, 12%) as an ammonium salt.

**Synthesis of 1c**

An aqueous solution of m^7^Gp_5_OC_3_H_3_ ammonium salt (0.65 mg, 0.81 μmol, 10 µL, 0.08 M) was mixed with a solution 5-FAM-azide (0.37 mg, 0.84 μmol) in DMSO (14 μL), followed by addition of 1.0 M aqueous solution of CuSO_4_∙5H_2_O (0.75 mg, 3.0 μmol, 3.0 μL) and 1.0 M sodium ascorbate (1.2 mg, 6.0 μmol, 6.0 μL). The resulting mixture was diluted with DMSO (20 μL) and vortexed at room temperature. After 6 h the reaction was quenched by addition of an aqueous 0.1 M solution of Na_2_EDTA (5.6 mg, 15 μmol, 150 μL). The mixture was centrifuged to remove excess of fluorescein dye, which precipitated from the solution. The product was purified by semi-preparative RP HPLC to give **1c** (0.50 mg, 0.4 μmol, 50%) as an ammonium salt.

**Synthesis of 1d**

Aqueous solution of m^7^GpCH_2_ppC_4_H_5_ ammonium salt (2.1 mg, 3.4 μmol, 20 μL, 0.17 M) was mixed with a solution of 6-FAM-azide (3.5 mg, 7.9 μmol) in DMSO (10 μL), followed by addition of 1.0 M aqueous solution of CuSO_4_∙5H_2_O (0.8 mg, 3.2 μmol, 3.2 μL) and 1.0 M sodium ascorbate (1.3 mg, 6.5 μmol, 6.5 μL). The resulting mixture was diluted with DMSO (60 μL) and vortexed at a room temperature. After 2.5 h the reaction was quenched by addition of Na_2_EDTA (1.2 mg, 3.2 μmol) and water (0.2 mL). The precipitated fluorescein dye was separated from the mixture by centrifugation. The product was purified by semi-preparative RP HPLC to give **1d** (1.4 mg, 1.3 μmol, 40%) as an ammonium salt

**Synthesis of 1e**

Aqueous solution of m^7^GTP_α_SOC_3_H_3_ D1 ammonium salt (1.5 mg, 2.3 μmol, 5 μL, 0.47 M) was mixed with a solution of 6-FAM-azide (1.5 mg, 3.4 μmol) in DMSO (40 μL), followed by addition of 1.0 M aqueous solution of CuSO_4_∙5H_2_O (0.4 mg, 1.5 μmol, 1.5 μL) and 1.0 M sodium ascorbate (0.6 mg, 3.0 μmol, 3.0 μL). The mixture was vortexed at a room temperature for 17 h and then the reaction was quenched by addition of Na_2_EDTA (1.9 mg, 5.0 μmol) and water (0.7 mL). The precipitated fluorescein dye was separated from the mixture by centrifugation. The product was purified by semi-preparative RP HPLC to give **1e** (0.5 mg, 0.46 µmol, 20%) as an ammonium salt.

**Synthesis of 1f**

Aqueous solution of m^7^GTP_α_SOC_3_H_3_ D2 ammonium salt (1.5 mg, 2.3 μmol, 5 μL, 0.47 M) was mixed with a solution of 6-FAM-azide (1.5 mg, 3.4 μmol, 0.08 M) in DMSO (40 μL), followed by addition of 1.0 M aqueous solutions of CuSO_4_∙5H_2_O (0.4 mg, 1.5 μmol, 1.5 μL) and 1.0 M sodium ascorbate (0.6 mg, 3.0 μmol, 3.0 μL). The mixture was vortexed at a room temperature for 17 h and then the reaction was quenched by addition of Na_2_EDTA (1.9 mg, 5.0 μmol) and water (0.7 mL). The precipitated fluorescein dye was separated from the mixture by centrifugation. The product was purified by semi-preparative RP HPLC to give **1f** (1.0 mg, 0.92 µmol, 40%) as an ammonium salt.

**Synthesis of 2a**

*N*^6^-L2_N_-AMP/TEAH^+^ (47 mg, 1250 mOD_260_, 0.080 mmol) and m^7^GDP-Im (50 mg, 915 mOD_260,_ 0.080 mmol) were suspended in DMSO (1.5 mL) and the anhydrous ZnCl_2_ (87 mg, 0.64 mmol, 8 equiv.) was added. The resulting solution was stirred at room temperature until RP HPLC analysis showed complete conversion of the *P*-imidazolide (ca. 8 h). Then, the reaction was quenched by addition of disodium EDTA (238 mg, 0.64 mmol, 8 equiv.) solution in water (15 mL) and adjusted to pH 7 with solid NaHCO_3_. The product was isolated by ion-exchange chromatography on DEAE-Sephadex using a linear gradient (0–0.9 M) of TEAB in deionized water as an eluent to give m^7^GpppA-*N*^6^-L2_N_ (65 mg, 1795 mOD_260_, 0.074 mmol, 92%) as a triethylammonium salt.

Triethylamine (8.4 µL, 0.06 mmol) and TSTU (18.2 mg, 0.06 mmol) were added to a 0.2 M solution of a 5(6)-carboxyfluorescein (20.7 mg, 0.055 mmol) in DMSO and the mixture was stirred at RT for 30 min. The resultant solution was added portion-wise (1 equivalent of dye every 15 minutes) to a solution of 0.1 M cap analogue in 0.5 M aqueous borate buffer pH 8.5. After each addition, the pH was monitored and re-adjusted to 8.5 using 0.5 M NaOH, if necessary. The reaction progress was monitored by RP HPLC. After complete conversion of the cap, the reaction mixture was diluted with water (4 mL), neutralized with 50% acetic acid and centrifuged. The precipitate containing unreacted dye was discarded and the products were isolated by semi-preparative RP HPLC to give single isomers of m^7^GpppA-*N*^6^-L2_N_-(6)FAM **2a** (166 mOD_490nm_, 2.45 µmol, 13%) and m^7^GpppA-*N*^6^-L2_N_-(5)FAM (320 mOD_490nm_, 4.71 µmol, 26%).

**Synthesis of 2b**

*N*^6^-L6_N_-AMP (163 mg, 3775 mOD_260_, 0.25 mmol) and m^7^GDP-Im (155 mg, 2865 mOD_260,_ 0.25 mmol) were suspended in DMSO (4.8 mL) and the anhydrous ZnCl_2_ (272 mg, 2.0 mmol, 8 equiv.) was added. The resulting solution was stirred at room temperature until RP HPLC analysis showed complete conversion of the *P*-imidazolide (ca. 8 h). Then, the reaction was quenched by addition of disodium EDTA (744 mg, 2.0 mmol, 8 equiv.) solution in water (50 mL) and adjusted to pH 7 with solid NaHCO_3_. The product was isolated by ion-exchange chromatography on DEAE-Sephadex using a linear gradient (0–0.9 M) of TEAB in deionized water as an eluent to give m^7^GpppA-*N*^6^-L6_N_ (152 mg, 3337 mOD_260_, 0.14 mmol, 56%) as a triethylammonium salt.

Triethylamine (7.7 µL, 0.055 mmol) and TSTU (16.7 mg, 0.055 mmol) were added to a 0.2 M solution of a 5(6)-carboxyfluorescein (19.0 mg, 0.05 mmol) in DMSO and the mixture was stirred at RT for 30 min. The resultant solution was added portion-wise (1 equivalent of dye every 15 minutes) to a solution of 0.1 M cap analogue in 0.5 M aqueous borate buffer pH 8.5. After each addition, the pH was monitored and re-adjusted to 8.5 using 0.5 M NaOH, if necessary. The reaction progress was monitored by RP HPLC. After complete conversion of the cap, the reaction mixture was diluted with water (5 mL), neutralized with 50% acetic acid and centrifuged. The precipitate containing unreacted dye was discarded and the products were isolated by semi-preparative RP HPLC to give single isomers of m^7^GpppA-*N*^6^-L6_N_-(6)FAM **2b** (306 mOD_490nm_, 4.5 µmol, 27%) and m^7^GpppA-*N*^6^-L6_N_-(5)FAM (740 mOD_490nm_, 4.71 µmol, 50%).

**Synthesis of 2c**

*N*^6^-L13_N_-AMP (183 mg, 3650 mOD_260_, 0.243 mmol) and m^7^GDP-Im (150 mg, 2770 mOD_260,_ 0.243 mmol) were suspended in DMSO (5 mL) and the anhydrous ZnCl_2_ (264 mg, 1.94 mmol, 8 equiv.) was added. The resulting solution was stirred at room temperature until RP HPLC analysis showed complete conversion of the *P*-imidazolide (ca. 24 h). Then, the reaction was quenched by addition of disodium EDTA (722 mg, 1.94 mmol, 8 equiv.) solution in water (50 mL) and adjusted to pH 7 with solid NaHCO_3_. The product was isolated by ion-exchange chromatography on DEAE-Sephadex using a linear gradient (0–0.9 M) of TEAB in deionized water as an eluent to give m^7^GpppA-*N*^6^-L13_N_ (179 mg, 3575 mOD_260_, 0.150 mmol, 62%) as a triethylammonium salt).

Triethylamine (12.7 µL, 0.091 mmol ) and TSTU (27.3 mg, 0.091 mmol) were added to a 0.2 M solution of a 5(6)-carboxyfluorescein (31.0 mg, 0.082 mmol) in DMSO and the mixture was stirred at RT for 30 min. The resultant solution was added portion-wise (1 equivalent of dye every 15 minutes) to a solution of 0.1 M cap analogue in 0.5 M aqueous borate buffer pH 8.5. After each addition, the pH was monitored and re-adjusted to 8.5 using 0.5 M NaOH, if necessary. The reaction progress was monitored by RP HPLC. After complete conversion of the cap, the reaction mixture was diluted with water (4 mL), neutralized with 50% acetic acid and centrifuged. The precipitate containing unreacted dye was discarded and the products were isolated by semi-preparative RP HPLC to give single isomers of m^7^GpppA-*N*^6^-L13_N_-(6)FAM **2c** (174 mOD_490nm_, 2.56 µmol, 12%) and m^7^GpppA-*N*^6^-L13_N_-(5)FAM (370 mOD_490nm_, 5.45 µmol, 26%).

**Synthesis of 2d**

Stock solutions of m^7^G^5’S^pppG_m_-L3_Y_/NH_4_^+^ (5.1 mOD_260nm_, 0.23 µmol, in 11 µL of water), (6)FAM-azide (0.10 mg, 0.23 µmol, in 5.6 µL of DMSO) and CuSO_4_/THPTA 1:1 (10 mM, 11 µL) were mixed and the reaction was initiated by addition of a freshly prepared sodium ascorbate stock solution (0.2M in water, 2.3 µL). After 2 h the reaction was quenched by addition of a 5 mg/mL solution of EDTA in water pH 6 (150 µL). An excess of dye was removed by extraction with ethyl acetate and the product was isolated from the aqueous phase by analytical RP HPLC to give after repeated freeze-drying from water an ammonium salt of m^7^G^5’S^pppG_m_-linker-FAM **2d** (3.9 mOD_490nm_, 0.058 µmol, 25%).

**Synthesis of 2e and 2f**

*N*^6^-L6_N_-AMP (332 mg, 0.61 mmol, TEAH^+^ salt), imidazole (413 mg, 6.08 mmol, 10 eq.), 2,2’‑dithiodipyridine (401 mg, 1.82 mmol, 3 eq.) and triethylamine (341 μL, 2.43 mmol, 4 eq.) were suspended in anhydrous DMF (5 mL). Then triphenylphosphine (478 mg, 1.82 mmol, 3 eq.) was added and the mixture was stirred at room temperature overnight. The product was precipitated by addition of a sodium perchlorate (324 mg, 3.04 mmol, 5 eq.) solution in anhydrous acetonitrile (40 mL). After cooling the precipitate was centrifuged, washed several times with cold acetonitrile by repeated centrifugation and dried in a vacuum desiccator to afford sodium salt of *N*^6^-L6_N_-AMP-Im (260 mg, 0.52 mmol, 85%).

*N*^6^-L6_N_-AMP-Im (80 mg, 2393 mOD_260_, 0.159 mmol) and m^7^GpCH_2_p (105 mg, 1817 mOD_260,_ 0.159 mmol) were suspended in DMSO (2.8 mL) an the anhydrous ZnCl_2_ (173 mg, 1.27 mmol, 8 equiv.) was added. The resulting solution was stirred at room temperature until RP HPLC analysis showed complete conversion of the *P*-imidazolide (ca. 5 h). Then, the reaction was quenched by addition of disodium EDTA (473 mg, 1.27 mmol, 8 equiv.) solution in water (28 mL) and adjusted to pH 7 with solid NaHCO_3_. The product was purified by ion-exchange chromatography on DEAE-Sephadex using a linear gradient (0–0.9 M) of TEAB in deionized water as an eluent to give m^7^GpCH_2_ppA-*N*^6^-L6_N_ (77.2 mg, 1690 mOD_260_, 0.071 mmol, 45%) as a triethylammonium salt).

Triethylamine (5.2 µL, 0.038 mmol) and TSTU (11.3 mg, 0.038 mmol) were added to a 0.2 M solution of a 5(6)-carboxyfluorescein (12.9 mg, 0.034 mmol) in DMSO and the mixture was stirred at RT for 30 min. The resultant solution was added portion-wise (1 equivalent of dye every 15 minutes) to a solution of 0.1 M cap analogue in 0.5 M aqueous borate buffer pH 8.5. After each addition, the pH was monitored and re-adjusted to 8.5 using 0.5 M NaOH, if necessary. The reaction progress was monitored by RP HPLC. After complete conversion of the cap, the reaction mixture was diluted with water (3 mL), neutralized with 50% acetic acid and centrifuged. The precipitate containing unreacted dye was discarded and the products were isolated by semi-preparative RP HPLC to give single isomers of m^7^GpCH_2_ppA-*N*^6^-L6_N_-(6)FAM **2e** (136 mOD_490nm_, 2.0 µmol, 17%) and m^7^GpCH_2_ppA-*N*^6^-L6_N_-(5)FAM **2f** (333 mOD_490nm_, 4.9 µmol, 43%).

**Synthesis of 2g**

Stock solutions of m^7^GpCH_2_ppG-2′-*O*-L3_Y_/NH_4_^+^ (22.5 mOD_260nm_, 1.1 µmol, in 56 µL of water), (6)‑FAM‑azide (0.50 mg, 1.1 µmol, in 28 µL of DMSO) and CuSO_4_/THPTA 1:1 (10 mM, 56 µL) were mixed and the reaction was initiated by addition of a freshly prepared sodium ascorbate stock solution (0.2M in water, 11 µL). After 3 h the reaction was quenched by addition of a 5 mg/mL solution of EDTA in water pH 6 (0.85 mL). An excess of dye was removed by extraction with ethyl acetate and the product was isolated from the aqueous phase by analytical RP HPLC to give after repeated freeze-drying from water an ammonium salt of m^7^GpCH_2_ppG-2′-*O*-linker-FAM **2g** (21 mOD_490nm_, 0.31 µmol, 27%).

**Synthesis of 2h**

Stock solutions of m^7^GpCH_2_ppG-3′-*O*-L3_Y_/NH_4_^+^ (5.1 mOD_260nm_, 0.23 µmol, in 11 µL of water), (6)‑FAM‑azide (0.10 mg, 0.23 µmol, in 5.6 µL of DMSO) and CuSO_4_/THPTA 1:1 (10 mM, 11 µL) were mixed and the reaction was initiated by addition of a freshly prepared sodium ascorbate stock solution (0.2M in water, 11 µL). After 2 h the reaction was quenched by addition of a 5 mg/mL solution of EDTA in water pH 6 (150 µL). An excess of dye was removed by extraction with ethyl acetate and the product was isolated from the aqueous phase by analytical RP HPLC to give after repeated freeze-drying from water an ammonium salt of m^7^GpCH_2_ppG-3′-*O*-linker-FAM **2h** (3.6 mOD_490nm_, 0.053 µmol, 23%).

**Synthesis of 2i**

Triethylammonium salt of m^7^GpCH_2_ppG-3′-*O*-L13_N_ (10 mOD_260nm_, 0.44 µmol) was dissolved in DMSO (8.8 µL) and (6)FAM-NHS (0.42 mg, 0.88 µmol) was added. The mixture was vortexed at RT overnight and the reaction was quenched by addition of water (100 µL). An excess of dye was removed by extraction with ethyl acetate and the product was isolated from the aqueous phase by analytical RP HPLC to give after repeated freeze-drying from water an ammonium salt of m^7^GpCH_2_ppG-3′-*O*-L13_N_-(6)FAM **2i** (7.5 mOD_490nm_, 0.10 µmol, 22%).

**Synthesis of 3a**

Triethylammonium salt of pApG dinucleotide (126 mOD_260nm_, 9.3 µmol) was dissolved in DMF (93 µL), followed by addition of imidazole (4.0 mg, 60 µmol), 2,2′-dithiodipyridine (2.5 mg, 11 µmol), triethylamine (2.6 µL, 19 µmol) and triphenylphosphine (4.8 mg, 18 µmol). The reaction mixture was stirred at RT for 24 h and the product was precipitated by addition of NaClO_4_ (5.7 mg, 46 µmol) solution in acetonitrile (930 µL). The solid was centrifuged, washed 3 times with cold acetonitrile and dried under reduced pressure to give a sodium salt of Im‑pApG that was immediately used for coupling reaction.

The Im‑pApG was suspended in DMSO (186 µL) and L6_N_-m^7^GDP/TEAH^+^ (5.6 mg, 7.0 µmol) was added, followed by anhydrous ZnCl_2_ (10.1 mg, 74.4 µmol). The mixture was stirred at RT for 19 h and the reaction was quenched by addition of EDTA (27.7 mg, 74.4 µmol) and NaHCO_3_ (13.8 mg, 165 µmol) in water (1.4 mL). The product was isolated by ion-exchange chromatography on DEAE Sephadex (linear gradient of 0 – 0.7 M TEAB) to give after evaporation a triethylammonium salt of a mixture of 2′‑*O*/3′‑*O* regioisomers of L6_N_-m^7^GpppApG (56 mOD_260nm_, 1.75 µmol, 38% from pApG) as a white solid.

Triethylammonium salt of L6_N_-m^7^GpppApG (54 mOD_260nm_, 1.70 µmol) was dissolved in DMSO (68 µL) and (5)FAM-NHS (1.6 mg, 3.40 µmol) was added. The mixture was vortexed at RT overnight and the reaction was quenched by addition of water (680 µL). An excess of dye was removed by extraction with ethyl acetate and the product was isolated from the aqueous phase by semi-preparative RP HPLC to give after repeated freeze-drying from water an ammonium salt of (5)FAM-L6_N_-m^7^GpppApG **3a** (42 mOD_495nm_, 0.63 µmol, 37%).

**Synthesis of 3b and 3c**

Triethylammonium salt of pA_m_pG dinucleotide (366 mOD_260nm_, 13.5 µmol) and 1,1′-carbonyldiimidazole (CDI; 37.2 mg, 230 µmol) were dissolved in DMSO (460 µL) and the mixture was heated for 20 min in the microwave reactor (P_max_ = 5W, T_max_ = 50±1 °C). Then, an excess of CDI was hydrolyzed with water (6.2 µL, 344 µmol) and 4,7,10-trioxatridecane-1,13-diamine (11.1 µL, 50.5 µmol) was added, followed by DBU (1.0 µL, 5.7 µmol). The reaction progress was monitored by RP-HPLC to avoid substitution of *P*-imidazolide by the amine. After completion of the linker attachment (ca. 3h), the *P*-imidazolide was precipitated from the reaction mixture by addition of LiClO_4_ (8.6 mg, 81 µmol) solution in acetonitrile (4.59 mL). The solid was centrifuged, washed 3 times with cold acetonitrile and dried under reduced pressure to give a lithium salt of Im‑pA_m_pG-L13_N_ that was immediately used for coupling reaction.

The Im‑pA_m_pG-L13_N_ was suspended in DMSO (270 µL) and m^7^GDP/TEAH^+^ (18.7 mg, 33.8 µmol) was added, followed by anhydrous ZnCl_2_ (36.7 mg, 270 µmol). The mixture was stirred at RT for 24 h and the reaction was quenched by addition of EDTA (126 mg, 338 µmol) and NaHCO_3_ (63 mg, 750 µmol) in water (6.3 mL). The product was isolated by ion-exchange chromatography on DEAE Sephadex (linear gradient of 0 – 0.7 M TEAB) to give after evaporation a triethylammonium salt of a mixture of 2′‑O/3′‑O regioisomers of m^7^GpppA_m_pG-L13_N_ (152 mOD_260nm_, 4.75 µmol, 35% from pA_m_pG) as a white solid.

A portion of m^7^GpppA_m_pG-L13_N_ (5.2 mOD_260nm_, 163 nmol) was dissolved in DMSO (6.5 µL) and a solution of (6)FAM-NHS (0.15 mg, 325 nmol) in DMSO (0.65 µL) was added. The mixture was vortexed at RT for 20 h and the reaction was quenched by addition of water (65 µL). An excess of dye was removed by extraction with ethyl acetate and the product was isolated from the aqueous phase and regioisomers were separated by analytical RP HPLC to give after repeated freeze-drying from water an ammonium salts of m^7^GpppA_m_pG-2′-O-L13_N_-(6)FAM **3b** (1.53 mOD_495nm_, 23 nmol, 14%) and m^7^GpppA_m_pG-3′-O-L13_N_-(6)FAM **3c** (2.43 mOD_495nm_, 36 nmol, 22%).

References:

1. Rydzik, A. M.; Lukaszewicz, M.; Zuberek, J.; Kowalska, J.; Darzynkiewicz, Z. M.; Darzynkiewicz, E.; Jemielity, J., Synthetic dinucleotide mRNA cap analogs with tetraphosphate 5',5' bridge containing methylenebis(phosphonate) modification. *Org Biomol Chem* **2009,** *7* (22), 4763-76.

2. Huang, F.; Wang, G.; Coleman, T.; Li, N., Synthesis of adenosine derivatives as transcription initiators and preparation of 5' fluorescein- and biotin-labeled RNA through one-step in vitro transcription. *RNA* **2003,** *9* (12), 1562-70.

3. Warminski, M.; Kowalska, J.; Jemielity, J., Synthesis of RNA 5'-Azides from 2'-O-Pivaloyloxymethyl-Protected RNAs and Their Reactivity in Azide-Alkyne Cycloaddition Reactions. *Org Lett* **2017,** *19* (13), 3624-3627.

4. Kasprzyk, R.; Starek, B. J.; Ciechanowicz, S.; Kubacka, D.; Kowalska, J.; Jemielity, J., Fluorescent Turn-On Probes for the Development of Binding and Hydrolytic Activity Assays for mRNA Cap-Recognizing Proteins. *Chemistry* **2019,** *25* (27), 6728-6740.

5. Wanat, P.; Walczak, S.; Wojtczak, B. A.; Nowakowska, M.; Jemielity, J.; Kowalska, J., Ethynyl, 2-Propynyl, and 3-Butynyl C-Phosphonate Analogues of Nucleoside Di- and Triphosphates: Synthesis and Reactivity in CuAAC. *Org Lett* **2015,** *17* (12), 3062-5.

6. Warminski, M.; Sikorski, P. J.; Warminska, Z.; Lukaszewicz, M.; Kropiwnicka, A.; Zuberek, J.; Darzynkiewicz, E.; Kowalska, J.; Jemielity, J., Amino-Functionalized 5' Cap Analogs as Tools for Site-Specific Sequence-Independent Labeling of mRNA. *Bioconjug Chem* **2017,** *28* (7), 1978-1992.

7. Sikorski, P. J.; Warminski, M.; Kubacka, D.; Ratajczak, T.; Nowis, D.; Kowalska, J.; Jemielity, J., The identity and methylation status of the first transcribed nucleotide in eukaryotic mRNA 5' cap modulates protein expression in living cells. *Nucleic Acids Res* **2020,** *48* (4), 1607-1626.
